# Prediction of breast cancer prognosis using gene set statistics provides signature stability and biological context

**DOI:** 10.1186/1471-2105-11-277

**Published:** 2010-05-25

**Authors:** Gad Abraham, Adam Kowalczyk, Sherene Loi, Izhak Haviv, Justin Zobel

**Affiliations:** 1 Department of Computer Science and Software Engineering, The University of Melbourne, Parkville 3010, VIC, Australia; 2 NICTA Victoria Laboratory, The University of Melbourne, Parkville 3010, VIC, Australia; 3 Department of Translational Research and Functional Genomics Unit, Jules Bordet Institute, Brussels, Belgium; 4 Department of Medical Oncology, Peter MacCallum Cancer Centre, East Melbourne, VIC 3002, Australia; 5 Metastasis Research Laboratory, Peter MacCallum Cancer Centre, East Melbourne, VIC 3002, Australia; 6 The Blood and DNA Profiling Facility, Baker IDI Institute, Prahran, VIC 3004, Australia; 7 Department of Biochemistry, School of Medicine, University of Melbourne, VIC 3010, Australia

## Abstract

**Background:**

Different microarray studies have compiled gene lists for predicting outcomes of a range of treatments and diseases. These have produced gene lists that have little overlap, indicating that the results from any one study are unstable. It has been suggested that the underlying pathways are essentially identical, and that the expression of gene sets, rather than that of individual genes, may be more informative with respect to prognosis and understanding of the underlying biological process.

**Results:**

We sought to examine the stability of prognostic signatures based on gene sets rather than individual genes. We classified breast cancer cases from five microarray studies according to the risk of metastasis, using features derived from predefined gene sets. The expression levels of genes in the sets are aggregated, using what we call a set statistic. The resulting prognostic gene sets were as predictive as the lists of individual genes, but displayed more consistent rankings via bootstrap replications within datasets, produced more stable classifiers across different datasets, and are potentially more interpretable in the biological context since they examine gene expression in the context of their neighbouring genes in the pathway. In addition, we performed this analysis in each breast cancer molecular subtype, based on ER/HER2 status. The prognostic gene sets found in each subtype were consistent with the biology based on previous analysis of individual genes.

**Conclusions:**

To date, most analyses of gene expression data have focused at the level of the individual genes. We show that a complementary approach of examining the data using predefined gene sets can reduce the noise and could provide increased insight into the underlying biological pathways.

## Background

Much attention has been given to predicting patient survival from microarray data. In breast cancer, van 't Veer et al. [1,2] set out to find genes that could be used to predict whether breast cancer patients would experience a metastasis five years after surgery (a binary variable). Their list of 70 genes (NKI70) performed well in predicting the clinical outcome (area under receiver-operating characteristic curve, AUC ≈ 0.7) and is currently commercially available as a prognostic test for breast cancer patients. However, Ein-Dor et al. [3] used the stratified bootstrap to show that many other gene lists of similar predictive ability could be found from the same data. Moreover, the overlap between the gene lists was low. Similarly, Michiels et al. [4] reported that gene lists derived from seven published cancer studies were highly unstable, and suggested random resampling for validation of such signatures. Many other studies of predicting breast cancer survival using gene expression have followed, with differing gene lists [5]. Reyal et al. [6] examined the performance of nine gene signatures on seven breast cancer datasets. Although the signatures had similar predictive ability, they showed little agreement on the individual predictions -- less than 50% of the individual predictions agreed -- and had only a small number of overlapping genes.

These results raise several questions. First, are these genes truly associated with cancer and metastasis, or are they spurious, the result of complex models overfitting the data? Second, if these genes are associated with cancer, are they also causally related to it? (a gene may be "down stream" of a cancer-causing gene and therefore be associated with but not cause cancer). Third, can a stable gene list be found at all? Fourth, do the different lists actually represent the same underlying pathways [7] and hence are in more agreement than is otherwise apparent? Interestingly, it is not clear whether there is any advantage in using gene lists, as opposed to single genes, at least from a prediction point of view -- Haibe-Kains et al. [8] found that classifiers built on single genes were as accurate (in terms of AUC) as classifiers based on multiple genes. Similarly, Lai et al. [9] reported that simpler models (univariable models) were more stable than multivariable models, possibly due to overfitting of the latter.

If the different predictive genes truly represent the same underlying biology, then perhaps what is needed is to evaluate genes as members of gene pathways, and use the pathway information to somehow guide the selection of predictive genes. Ideally, one would like to have detailed gene pathway information, which can then be used to select genes with a potential causal link to cancer and metastasis. This has largely not been possible due to limitations on data size (too few samples) and the complexity of gene interactions. Therefore, the problem of finding the pathway information must be tackled in other ways. One way is to assume that genes with correlated expression belong together in one pathway (or are somehow otherwise related to each other, even if they do not interact directly), and to find the sets *de novo *in the data, using methods such as searching over a space of models representing regulation programs [10] and *k*-means clustering [11]. Similarly, van Vliet et al. [12] used an unsupervised module discovery method to find gene modules, calculated a discrete module activity score, and used the score as feature for a naïve Bayes classifier. They reported that classifiers based on gene sets were slightly better predictors of breast cancer outcome than those based on individual genes. Chuang et al. [13] used a mutual-information scoring approach to analyse known protein-protein interaction (PPI) networks, infer gene pathways, and find subnetworks predictive of breast cancer metastasis.

The other main approach has been to use external pathway information, for example, from the literature. Svensson et al. [14] analysed expression data from ovarian cancers based on gene sets from the Gene Ontology (GO) [15]; to represent the set's expression they used a statistic that is essentially a majority-vote of the over- and under-expressed genes (whether the set is over- or under-expressed on average). In a large study of 12 breast cancer datasets, Kim and Kim [16] reported a classification accuracy of 67.6% over 6 additional datasets, using 2411 gene sets from GO categories, pathway data, and other sources. They, too, reported low overlap between the top gene sets identified, in terms of their common genes. Lee et al. [17] used the MSigDB C2 gene sets, selected gene sets using the *t*-test on their constituent genes, and used the sets as features for classification in several cancer datasets, including breast cancer. They, did not, however, examine whether features derived from gene sets are any more stable than those based on individual genes, a question which is the focus of our work.

Once a tentative or known gene pathway has been identified, the next issue is how to use the expression levels of its constituent genes in a meaningful way. Some options are to use the mean or median expression [18], the first few principal components [19], and the *z*-statistic [20]. Below we examine several approaches, which we call *set statistics*.

### Our Approach

In this work we propose using prior knowledge, in the form of pre-specified gene sets from the Molecular Signatures Database (MSigDB) [21] dataset, in order to form new features from the individual genes. Moving away from considering genes in isolation, these features serve as proxies for measuring the activity of the set as a whole. There are many approaches to *gene set enrichment *[22]; however, it is not clear whether these enrichment measures imply good predictive abilities. In contrast, we compare features derived from gene sets with features based on individual genes, with respect to the following criteria:

• discrimination: ability to predict metastasis within 5 years, both on average and its variance;

• stability of the ranks of individual features within datasets;

• concordance between the weights and ranks of features from different datasets;

• and the underlying biological process pointed to by the features.

## Methods

We explored a range of methods for extracting gene sets. These statistics are described below; we first discuss the data used.

### Data

We used five breast cancer datasets from NCBI GEO [23]: GSE2034 [24], GSE4922 [25], GSE6532 [26,27], GSE7390 [28], and GSE11121 [29]. All five are Affymetrix HG-U133A microarrays (some datasets include other platforms; these platforms were excluded). We removed quality control probesets, probesets with close to zero variance across samples, and probesets with more than 15% missing expression levels (otherwise, missing values were imputed using the median expression of the non-missing samples [[30], pp. 48-50]). In total, each microarray had 22,215 remaining probesets. The datasets were independently normalised (see Additional File 1).

#### Data Composition

The data contains both lymph-node-negative and node-positive breast cancer patients. For GSE7390, GSE11121, and GSE2034, none of the patients received adjuvant treatment. For GSE6532 and GSE4922, some patients received adjuvant therapy; these were removed from the data. The data contains patients with both ER-positive and ER-negative tumours. Patients were classified into two groups, low and high risk, according to the time to distant metastasis, using a cutoff point of 5 years. Patients censored before the cutoff were considered noninformative and were removed from the data, as shown in Table 1.

**Table 1:** Sample sizes and breakdown by class

Dataset	Good Obs.			Removed Obs.
	< 5 years	≥ 5 years	Total	
GSE2034	82	165	247	8
GSE4922	30	103	133	9
GSE6532	21	91	112	25
GSE7390	36	154	190	8
GSE11121	28	154	182	18

Observations (samples) were removed if they were censored before the 5-year cutoff.

### Gene Sets

We used five MSigDB http://www.broadinstitute.org/gsea/msigdb gene set collections: C1 (positional gene sets), C2 (curated gene sets), C3 (motif gene sets), C4 (computational gene sets), and C5 (GO terms) [21], a total of 5452 gene sets, of which 5414 sets could be mapped to the Affymetrix HG-U133A probesets. Note that the C2 collection includes sets derived from KEGG [31] and Gene Ontology [15], among others.

### Set Statistics

The purpose of the set statistic is to reduce the set's expression matrix to a single vector, which is then used as a feature for classification. The intention is for the set statistic to be representative of the expression levels of the set, in a useful way. Here we describe the different set statistics used in this work. All of our set statistics are *unsupervised*, in the sense that they do not take into account the metastatic class, unlike methods such as the *t*-test [32], GSEA [21], or GSA [33]; any standard classifier, such as a support-vector machine (SVM), can be built on top of these features.

#### Mathematical Notation

Here, **X **is the *p *× *N *matrix of gene expression levels, where *p *denotes the number of genes and *N *denotes the number of samples. Every gene belongs to one or more gene sets , such that  ∈ {1, ..., *p*}, *j *= 1, ..., *M*, where *M *is the number of gene sets. *s*_*j *_= || and *s*_¬*j *_denote the number of genes inside and outside the *j*th set, respectively.

#### Set Centroid and Set Median

The centroid is simply the mean expression level over all genes in the set. The matrix of all centroids is an *M *× *N *matrix with entries(1)

where *x*_*ki *_is the expression level for the *k*th gene in the *i*th sample. Similarly, the set median is the median expression level for all genes in the set, for a given sample.

The motivation for the centroid is that it reduces the variance of noise in the feature, since the sample variance of the mean of a random vector ***x ***= (*x*_1_, ..., *x*_*n*_) is the square of the standard error of the sample mean ,(2)

for *n *> 1. The actual decrease in variance depends on the degree of correlation of the variables. Another interpretation is that all the genes in the same set are effectively shrunk towards the set mean, thereby reducing the effect of outlier genes [32] and reducing overfitting. The set median is similar to the centroid, except that it is less sensitive to outliers.

#### Set Medoid

The matrix of medoids is defined as the gene in the set *S*_*j *_closest (in Euclidean distance) to the centroid, for each sample *i*(3)

where *x*_*ki *_is the expression level for the *k*th gene (out of the *s*_*j *_genes in the set) in the *i*th sample. In this formulation, the set medoid is not the same as the set median.

#### Set *t*-Statistic

The set centroid does not take into account different means and variances between the genes, nor the fact that a gene may have a high mean but high variance as well (low signal-to-noise ratio). An alternative is to use the one-sample *t*-statistic. The matrix of *t*-statistics is computed by first centering and scaling the expression matrix so that each gene has mean zero and unit variance, and then computing the *t*-statistic for each set in each sample,(4)

where centroid_*ji *_is the centroid statistic from Eq. 1, and sd_*ji *_is the standard deviation of the genes in set *j *in the *i*th sample. Scaling is done to prevent spurious *t*-statistics, due to very small variances, from inflating the importance of "non-interesting" genes. We excluded sets with fewer than 30 genes.

#### U-statistic p-value

The competitive U-statistic for the set, also known as Wilcoxon's rank-sum statistic [35], compares the mean rank of the genes *in the set *to the mean rank of the genes *outside the set*, for all samples. The U-statistic is calculated as follows.

1. Create a list of gene expression ranks  of the *s*_*j *_genes in the set in the *i*th sample.

2. Sum the ranks for the set 

3. The U-statistic for set *S*_*j *_in sample *i *is then *U*_*ij *_= *R*_*ij *_- *s*_*j*_(*s*_*j *_+ 1)/2.

For large *n*, the *j*th U-statistic is approximately normally-distributed, with *μ *= *s*_*j*_*s*_*¬j*_/2 and *σ*^2 ^= *s*_*j*_*s*_¬*j*_(*s*_*j *_+ *s*_¬*j *_+ 1)/12. Once the U-statistic is computed, we use its log *p*-value as the feature for the classifier.

This statistic is slightly unusual since it pits gene sets against other gene sets, that is, its distribution depends on the number of genes sets rather than samples. Goeman and Bühlmann [36] argue that this statistic is inappropriate since it switches the standard relationship between genes and samples in the experimental setup (the sample size becomes the number of genes, not the number of microarrays); however, Barry et al. [37] consider it a useful statistic nonetheless. In any case, we use this statistic only as a feature for a classifier, and not to directly make inferences about the statistical significance of the sets' expression levels.

#### 1st Principal Component of the Set

Principal Component Analysis (PCA) is performed using the singular value decomposition (SVD) of the gene set's *s*_*j *_× *N *expression matrix **X**_*j*_, defined as(5)

where **U**_*j *_and **V**_*j *_are matrices whose columns are the left and right singular vectors, respectively, and **D**_*j *_is a diagonal matrix with the diagonal being the eigenvalues (also called loadings).

The first eigenvector **ν**_1*j *_(first column of **V**_*j*_) explains the highest amount of variance in . The 1st principal component (PC) of the matrix, PC1_*j*_, is obtained by projecting the data onto that eigenvector(6)

where **ν**_1*j *_is an *s*_*j *_× 1 column vector. Hence, PC1 is the best rank-1 approximation of the data. We mean-centred and scaled the matrix **X**^*T *^before applying PCA.

### The Centroid Classifier

Feature instability, manifested as discordant gene lists, can be caused both by inherent instability and by overfitting the classifier to the data. Therefore, to reduce the risk of overfitting, we use the centroid classifier [[38], pp. 4-6]. The centroid classifier is equivalent to a heavily-regularised support vector machine [39] and to Fisher Linear Discriminant Analysis (LDA) with diagonal covariance and uniform priors [40,41]. Therefore, we expect that it is less prone to overfitting than an SVM or similar classifiers. We further stabilise the centroid's estimate by averaging them over random subsamples of the data, see discussion of "Internal and External Validation."

The centroid classifier finds the *centroid *of each class, that is, the *p*-vector of average gene expression in each class. New observations are classified by comparing their expression with the two centroids, and choosing the closest centroid. Given a *p *× *N *matrix **Z**, the centroids (*p*-vectors) of the positive and negative classes are, respectively,(7)

where *n*^+ ^and *n*^- ^are the number of samples in each class, and *z*_*i *_is the *i*th expression vector of *p *features (*i*th column of **Z**). The centroid classifier predicts using the inner-product rule  = ⟨*z*_*i *_- *c*, *w*⟩, where *c *= (*c*^+ ^+ *c*^-^)/2 is the point midway between the centroids, and the weight of each feature is(8)

where *w *is the *p*-vector connecting the two centroids. The sign of  is then the predicted class. For calculating AUC we use  as the prediction, since it produces an AUC estimate with lower variance than using the class prediction sign ().

This centroid classifier is similar but not identical to the classifier used by van 't Veer [1]; they assigned each sample to the class that has the highest Pearson correlation of its centroid with the sample. This is equivalent to our version of the centroid classifier when the samples are scaled to unit norm [[42], p. 203]. See Additional File 1 for further discussion.

Despite its simplicity, the centroid classifier performs well in microarray studies [39] where commonly the number of features is much greater than the number of samples (*p *≫ *N*). For the centroid classifier, we observed discrimination similar to or better than several other classifiers (support vector machine, nearest shrunken centroids [40], and the van 't Veer [1] classifiers, see Additional File 1 for details). The centroid classifier requires no tuning, making it fast to compute, and making *nested *cross-validation unnecessary during feature selection (see [43] for examples of where it is required).

### Internal and External Validation

Since we have five datasets, it might be reasonable to combine them. However, we were interested in measuring the concordance *between *datasets rather than performing a meta-analysis. The inter-dataset analysis emulates the real-world situation where different studies are performed separately, rather than pooled together. Therefore, we distinguish between *internal *and *external *validation. In the former, we estimate the classifier's generalisation *inside *each dataset, using repeated random subsampling; the subsampling is used to form a *bagged classifier *for each dataset (see below). We then perform external validation, where the bagged classifier from each dataset is used to predict the metastatic class of patients from another dataset. This is a more realistic estimate of the classifier's discriminative ability.

In the internal validation, we used repeated random subsampling to estimate the classifier's internal generalisation error, as measured by AUC (see Additional File 1 for the AUC from internal validation). In this approach, the dataset is randomly split *B *times into training and testing parts (2/3 and 1/3 of the data, respectively). We used *B *= 25 splits. Repeated random subsampling with a 2/3-1/3 split is similar to the 0.632 bootstrap without replacement [44]. Each split results in one model; the predictions from *B *models are then combined into one prediction -- *bagging *[[45], pp. 246-250] -- by averaging over the *B *predictions , and using that vector of averages as the final prediction. Bagging also reduces the variance of the predictions, without increasing the bias [45].

## Results and Discussion

### Classification

We observed that the discrimination from the internal and external validation were similar, showing that the centroid classifier did not significantly over- or under-fit the data. Following are results for the external validation; see Additional File 1 for the internal validation results.

**Figure 1:**
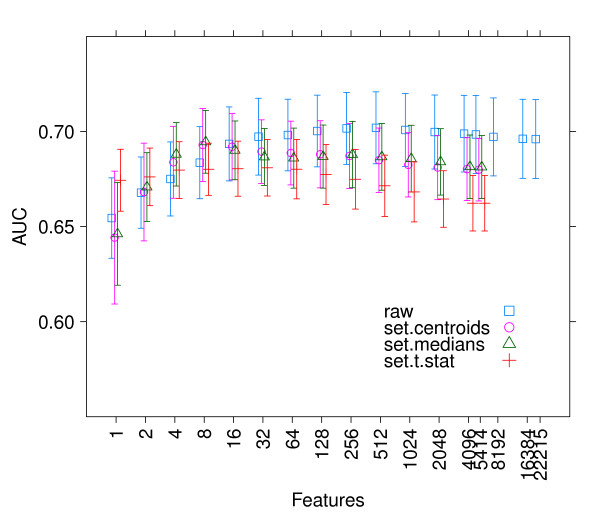
**Classification**. Average and 95% confidence intervals for AUC from external validation between the five datasets (*n *= 2 ×  = 20 (train, test) pairs) for different numbers of features. Note that each dataset ranks its features independently, hence, the *k*th feature is not necessarily the same across datasets. *raw *denotes individual genes.

Figure 1 shows AUC for external validation (trained on one dataset and predicted on another, a total of 2 = 20 predictions), using centroid classifiers trained on different numbers of features. The maximum number of features is 22,215 for genes and for 5414 gene sets. For clarity, we only show the results for classifiers based on expression of individual genes (denoted "raw"), the set centroid, the set median, and set *t*-statistic. (See Additional File 1, Figure 6 for more set statistics and Additional Table 1 for significance tests.) Unlike classifiers such as logistic regression or SVMs, the centroid classifier's weight of one feature does not depend on the others. While it is known that genes are not independently expressed, this strong assumption does not appear to reduce classification accuracy in our datasets. In addition, this assumption makes recursive feature elimination especially simple, since features can be eliminated in reverse order of their rank, where the rank is the absolute value of their weights *w*, and the rank does not need to be recomputed each time. The best AUC of about 0.7 is consistent with previous results based on either lists of individual genes [1,24] or of gene sets [12]. The set centroid, set medoid, set median, and set *t*-statistic showed similar AUC to that of individual genes. The set PC, and set U statistic showed statistically-significant reductions in AUC compared with individual genes, see Additional Table 1. (Note that each dataset ranks the sets independently, hence the top sets may be different. A consensus list of top sets is provided in Table 2).

**Table 2:** Top gene sets by average rank

#	Set	Cat.	Sign	MSigDB Description	Enriched GO BP Terms (adj. *p*-value)
1	GNF2_MKI67	C4	-1	Neighborhood of MKI67	"phosphoinositide-mediated signaling": 1.95 × 10^-10^, "spindle organization": 5.86 × 10^-6^, "establishment of mitotic spindle localization": 1.10 × 10^-5^, "kinetochore assembly": 5.48 × 10^-5^, "mitotic chromosome condensation": 1.37 × 10^-4^, "protein complex localization": 2.55 × 10^-3^, "regulation of striated muscle development": 2.55 × 10^-3^, "metaphase plate congression": 2.55 × 10^-3^
2	GNF2_CCNA2	C4	-1	Neighborhood of CCNA2	"phosphoinositide-mediated signaling": 4.05 × 10^-16^, "DNA replication": 1.04 × 10^-9^, "mitotic chromosome condensation": 1.32 × 10^-8^, "regulation of striated muscle development": 3.76 × 10^-3^, "metaphase plate congression": 3.76 × 10^-3^
3	GNF2_TTK	C4	-1	Neighborhood of TTK	"phosphoinositide-mediated signaling": < 2.22 × 10^-16^, "mitotic chromosome condensation": 4.35 × 10^-14^, "DNA replication": 1.01 × 10^-12^, "spindle organization": 1.37 × 10^-9^, "establishment of mitotic spindle localization": 9.59 × 10^-5^, "kinetochore assembly": 4.76 × 10^-4^, "DNA repair": 5.78 × 10^-3^, "mitosis": 9.44 × 10^-3^
4	GNF2_HMMR	C4	-1	Neighborhood of HMMR	"phosphoinositide-mediated signaling": < 2:22 × 10^-16^, "mitotic cell cycle spindle assembly checkpoint": 1.26 × 10^-11^, "spindle organization": 4.89 × 10^-10^, "mitotic chromosome condensation": 8.46 × 10^-8^, "cell proliferation": 6.22 × 10^-6^, "DNA replication": 1.09 × 10^-5^, "establishment of mitotic spindle localization": 5.33 × 10^-5^, "kinetochore assembly": 2.65 × 10^-4^, "protein complex localization": 8.29 × 10^-3^, "regulation of striated muscle development": 8.29 × 10^-3^, "metaphase plate congression": 8.29 × 10^-3^
5	GNF2_CDC20	C4	-1	Neighborhood of CDC20	"phosphoinositide-mediated signaling": < 2.22_10^-16^, "spindle organization": 2.20 × 10^-12^, "mitotic cell cycle spindle assembly checkpoint": 4.07 × 10^-11^, "mitotic chromosome condensation": 1.52 × 10^-9^, "cell proliferation": 8.96 × 10^-9^, "mitosis": 1.83 × 10^-8^, "establishment of mitotic spindle localization": 8.95 × 10^-5^, "kinetochore assembly": 4.45 × 10^-4^, "DNA replication": 7.83 × 10^-3^
6	GNF2_SMC2L1	C4	-1	Neighborhood of SMC2L1	"mitotic cell cycle spindle assembly checkpoint": 5.15 × 10^-13^, "mitotic chromosome condensation": 7.16 × 10^-9^, "phosphoinositide-mediated signaling": 2.14 × 10^-6^, "establishment of mitotic spindle localization": 1.31 × 10^-5^, "kinetochore assembly": 6.51 × 10^-5^, "protein complex localization": 2.90 × 10^-3^, "DNA strand elongation during DNA replication": 2.90 × 10^-3^, "regulation of striated muscle development": 2.90 × 10^-3^, "metaphase plate congression": 2.90 × 10^-3^, "cell proliferation": 2.94 × 10^-3^, "nucleotide-excision repair, DNA gap filling": 3.56 × 10^-3^
7	GNF2_H2AFX	C4	-1	Neighborhood of H2AFX	"cell proliferation": 9.28 × 10^-10^, "phosphoinositide-mediated signaling": 5.54 × 10^-7^, "mitosis": 8.48 × 10^-5^, "mitotic cell cycle spindle assembly checkpoint": 1.33 × 10^-4^, "protein complex localization": 1.63 × 10^-3^
8	GNF2_ESPL1	C4	-1	Neighborhood of ESPL1	"phosphoinositide-mediated signaling": 5.38 × 10^-11^, "kinetochore assembly": 3.12 × 10^-5^, "mitotic chromosome condensation": 6.75 × 10^-5^, "spindle organization": 7.76 × 10^-4^, "protein complex localization": 1.67 × 10^-3^, "regulation of striated muscle development": 1.67 × 10^-3^, "metaphase plate congression": 1.67 × 10^-3^
9	GNF2_RRM2	C4	-1	Neighborhood of RRM2	"phosphoinositide-mediated signaling": 4.52 × 10^-15^, "mitotic cell cycle spindle assembly checkpoint": 1.17 × 10^-9^, "spindle organization": 1.20 × 10^-7^, "DNA replication": 5.42 × 10^-6^, "cell proliferation": 1.97 × 10^-5^, "establishment of mitotic spindle localization": 4.09 × 10^-5^, "kinetochore assembly": 2.03 × 10^-4^, "protein complex localization": 6.80 × 10^-3^, "regulation of striated muscle development": 6.80 × 10^-3^, "metaphase plate congression": 6.80 × 10^-3^
10	GNF2_PCNA	C4	-1	Neighborhood of PCNA	"phosphoinositide-mediated signaling": < 2.22 × 10^-16^, "DNA replication": 1.47 × 10^-15^, "mitotic chromosome condensation": 2.36 × 10^-7^, "spindle organization": 4.33 × 10 ^-7^, "establishment of mitotic spindle localization": 9.59 × 10^-5^, "cell proliferation": 4.18 × 10^-4^, "DNA repair": 4.33 × 10^-4^, "kinetochore assembly": 4.76 × 10^-4^, "mitosis": 9.44 × 10^-3^

Top 10 gene sets by average rank over the five datasets, using the set centroid statistic. GO enrichment *p*-values are from a Bonferroni-adjusted one-sided Fisher's exact test (30,330 tests). Sign = -1 if expression is negatively associated with long-term survival, and vice versa. The background list for the test includes all Affymetrix HG-U133A probesets that could be mapped to GO BP terms, excluding IEA annotations.

**Figure 2:**
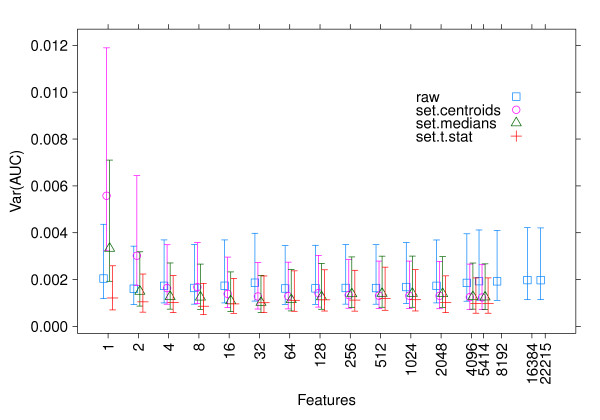
**Classification variance**. Variance and 95% confidence intervals of the AUC from external validation between the five datasets (*n *= 2 ×  = 20 (train, test) pairs) for different numbers of features. The confidence intervals are , where  is the *α *= 0.05 quantile for a chi-squared distribution with *n *- 1 degrees of freedom, *s*^2 ^is the sample variance and.

While AUC does not seem to improve, on average, by using set statistics rather than individual genes, Figure 2 shows that the *variance *of the AUC is consistently lower for the set *t*-statistic than for individual genes. This observation is consistent with the greater stability of gene set features, discussed below.

### Stability of Feature Ranks

We were interested in how the ranks of a single feature vary, since we prefer features that are highly ranked on average and have small variability about that average. If a feature has low average rank and large variability, it may sometimes appear at the top of list simply by chance when the experiment is repeated, indicating that it is not a reliable predictor. Features with high average rank and large variability may appear to be good predictors (on average) but will create unstable gene lists, manifesting as different datasets producing different gene lists of similar predictive ability.

To evaluate the variability of the ranks, we used the percentile bootstrap to sample the observations with replacement, generating a bootstrap distribution for the centroid weights for genes and gene sets in one dataset (GSE4922). Since there are 22,215 genes and only 5414 gene sets, a reduced gene list was derived by training a centroid classifier on the GSE11121 dataset and selecting the 5414 genes with the highest absolute centroid weights; the list was fixed across bootstrap replications.

**Figure 3:**
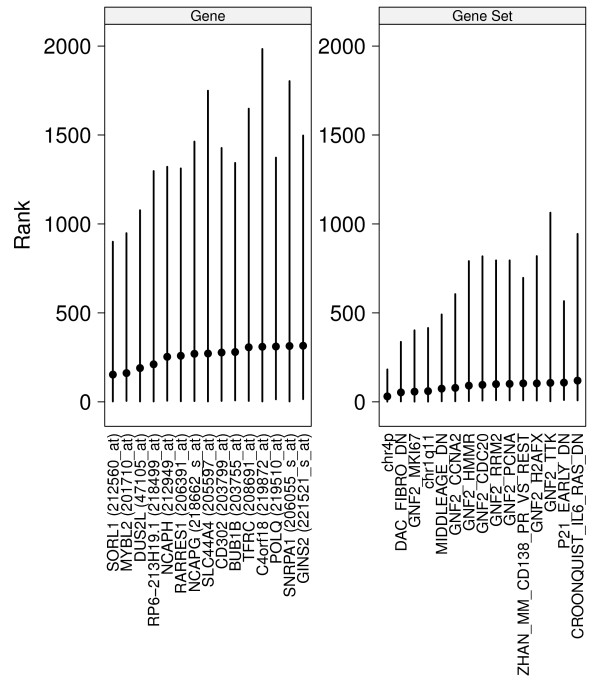
**Bootstrap**. Mean and 2.5%/97.5% of the ranks of genes and gene sets (set centroid statistic), over 5000 bootstrap replications of the GSE4922 dataset. The features have been sorted by their mean rank.

In many cases we are interested in a small signature comprised of the most useful or predictive features. Therefore, we selected the top 15 genes and gene sets based on their mean rank. Figure 3 shows the mean, 2.5%, and 97.5% percentiles from 5000 bootstrap replications, for these top features (shown from highest to lowest) using the set centroid statistic (see Additional Figure 8 for the results for other set statistics). It is clear that the top gene sets have lower variation than the top genes. In light of these results, it is not surprising that lists of prognostic genes show little overlap, as even the best ranked genes vary considerably within the same dataset, let alone between them; gene set features are more stable.

### Concordance of Datasets

We were interested in how the different datasets agreed on the importance of the features (genes or gene sets). We used two approaches: rank-correlation of the centroid classifier's weights, and concordance of the gene lists. For this section, the classifier was not bagged -- we trained a single centroid classifier on each dataset. We note that the datasets are independently normalised -- we are interested in the agreement between datasets *despite *different normalisation schemes.

**Figure 4:**
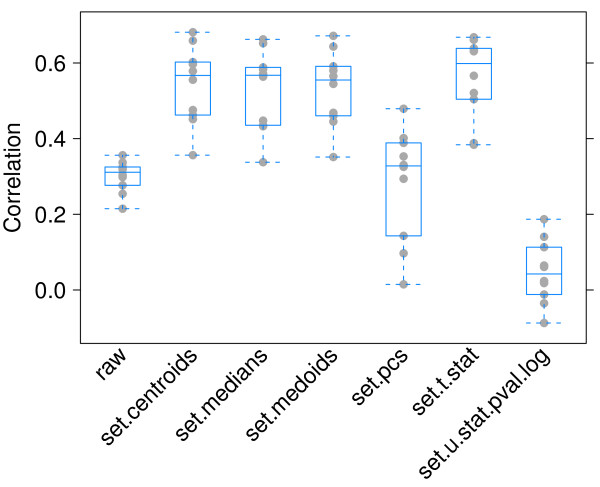
**External validation**. Spearman rank-correlation of the centroid classifier's weights from the five datasets (*n *= 10 comparisons). *raw *denotes individual genes.

We measured concordance using Spearman rank-correlation between the classifier weights estimated from each dataset, a total of  = 10 comparisons. Figure 4 shows the Spearman rank-correlations for each set statistic. It is evident that the rank-correlations for the weights of the set centroids, set median, set medoid, and the set *t*-statistic are higher than for the individual genes. This indicates that classifiers built from features based on gene sets are more stable than those built using individual genes, and are less likely to overfit.

**Figure 5:**
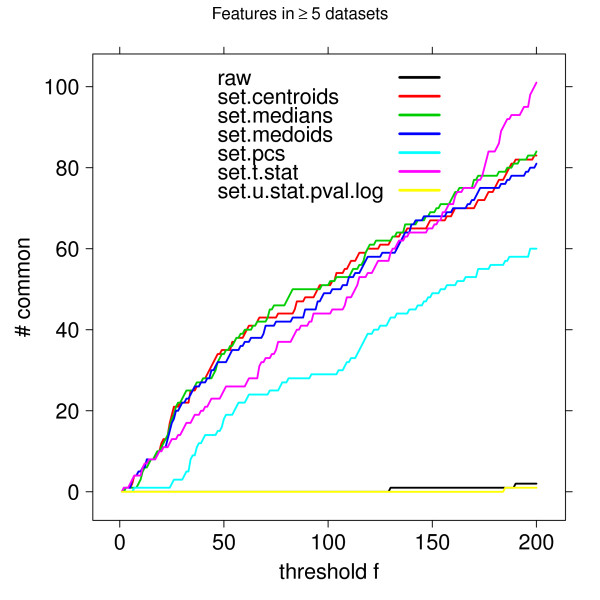
**List concordance**. Concordance of feature lists (genes or gene sets) for different cutoffs *f *= 1,...,200, counting the number of features occurring in all of the five datasets' lists, ranked higher than *f*. *raw *denotes individual genes.

To measure how the ranked lists produced by each dataset agreed on the top-ranked genes, we used the following approach. The features for each dataset were ranked by the absolute value of their *w *weight. Then, for each number *f *of features, *f *= 1, ..., *p*, we chose each dataset's top *f*-ranked features. Next, we counted how many of these *f *features occurred at least *k *of the five datasets. Results for *k *= 5 are shown in Figure 5. Lists based on individual genes show little overlap for cutoffs up to about 130 -- in other words, there are no genes that occur in all five datasets up to that cutoff. In comparison, the set statistics, especially the set medians and the set centroids, produce lists with higher overlap, even at cutoffs below 10. This result further supports the conclusion that lists of individual genes are highly unstable, and that the little overlap between reported prognostic signatures is to be expected.

### MSigDB Sets

Table 2 shows the top 10 gene sets by rank, where the rank was averaged over the feature ranks from the five datasets, using the set centroid statistic. Also shown is enrichment for BO Biological Process (BP) terms from a Bonferroni-adjusted Fisher's exact test, for the genes belonging to these sets. The top sets are enriched for GO BP terms related to the cell cycle and cell division processes, and for the PI3K pathway which interacts with the Ras oncogene [46], confirming the cell cycle process as one of the major biological mechanisms associated with breast cancer metastasis [47,48]. The top set, GNF2_MKI67, is related to Ki-67, a known marker of cancer proliferation [49].

The potential advantage of gene sets signatures over individual gene signatures depends on the degree of these genes' coexpression. A critical aspect of this performance, therefore, is the source for the grouping of genes into sets. The MSigDB is composed of five set classes depending on the annotation used to define the sets. Whereas categories C1 and C3 are derived from the chromosomal location and sequence of regulatory elements, respectively, categories C2 and C4 both originate from expression profiles; C5 is based on GO categories. In addition, the datasets these categories are based on vary with respect to sample size; whereas C4 was is based on hypothesis-free examination of co-expression across almost two thousand expression profiles, C2 is mainly based on publications of expression profiles, rarely using more than dozens of samples.

To see whether different MSigDB categories were more useful for predicting metastasis, we combined four datasets (GSE2034, GSE4922, GSE6532, and GSE7390) into a single training set. A separate centroid classifier was trained on each gene set, using the set-centroid statistic, and the gene sets were then ranked by their centroid classifier weights (negative to positive). We then tested the classifiers on the fifth dataset, GSE11121. Finally, we used the two-sample Kolmogorov-Smirnov statistic to compare the ranks from the different categories (see Additional File 1 for details and results for other set statistics).

**Figure 6:**
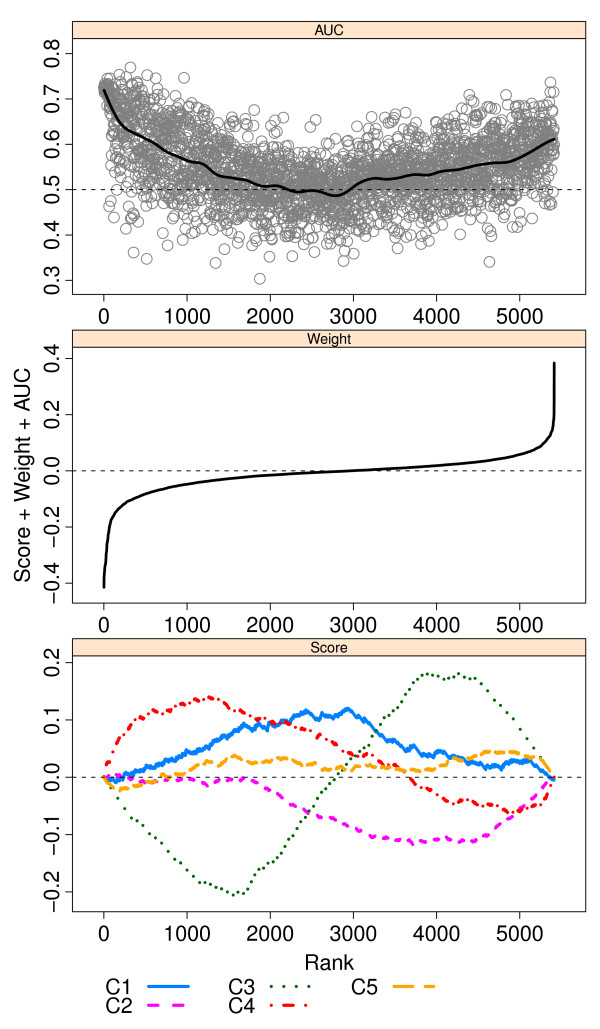
**Kolmogorov-Smirnov analysis**. Kolmogorov-Smirnov enrichment for MSigDB categories, using the set-centroid statistic. **A **AUC and spline smooth for each set, tested on GSE11121. **B **Number of mapped probesets in each set, on log_2 _scale, and spline smooth. **C **Two-sample Kolmogorov-Smirnov Brownian-bridge for each MSigDB category (*p*-values: C1: 1.44 × 10^-4^, C2: 3.55 × 10^-15^, C3: < 2.22 × 10^-16^, C4: 4.22 × 10^-13^, C5: 2.38 × 10^-2^).

Figure 6 shows the cumulative-sum statistic, from which the Kolmogorov-Smirnov statistic is computed, for the ranked gene sets. In order to link that rank with performance in sample classification, we plotted the classifier's AUC value for each of these sets along the rank. The results show that the C4 sets tend have extreme centroid weights, especially towards the negative side, than the other categories. In contrast, C2 sets show a concentration towards the positive weights, albeit much smaller. Category C3 tends to be concentrated in the middle ranks, and category C1 tends to be concentrated in the negative to middle ranks. Finally, category C5 is distributed more uniformly across the ranks; this may be since GO sets do not take the direction of expression change into account, potentially leading to sets composed of genes with a mixture of positive and negative correlations.

One problem with the set-centroid statistic is that for small sets, there is a higher probability of observing an extreme statistic by chance, since the variance of the sample mean decreases with set size. This implies that spurious set centroids (high absolute value) would be more common in smaller sets, leading to a bias towards smaller sets when ranking the sets. However, there does not seem to be a monotonic relationship between log-size and rank (see Additional File 1, Figure 10). Additionally, there is reasonable concordance between the sets as independently ranked by the five datasets. We conclude that while spurious effects due to the set-size cannot be ruled out, they does not seem to be a major factor in the set's rank. In any case, as an alternative to the set centroid, the set *t*-statistic can be used.

### Prognostic Gene Sets in Breast Cancer Molecular Subtypes

Breast cancer is a heterogeneous disease, with gene expression segregating the cases into different biological and clinically relevant subgroups, perhaps implying differing biological mechanisms for tumour growth and progression and suggesting separate cells of origin. It is reasonable to ask the question whether there is different aetiology related to breast cancer progression. The common molecular classification describes five classes -- basal-like, luminal A and B, normal breast tissue-like, and ERBB2+ [50]. Our results above show a strong cell-cycle signature as highly prognostic, supporting existing data [51]. The association of cell-cycle genes to increased risk of metastasis has been mainly attributed to the breast cancer cases that are ER+ [26,52], which comprise the majority of the breast cancer population. Therefore, we explored whether different signatures could be found by removing such cases and training the classifiers on the remaining samples.

**Figure 7:**
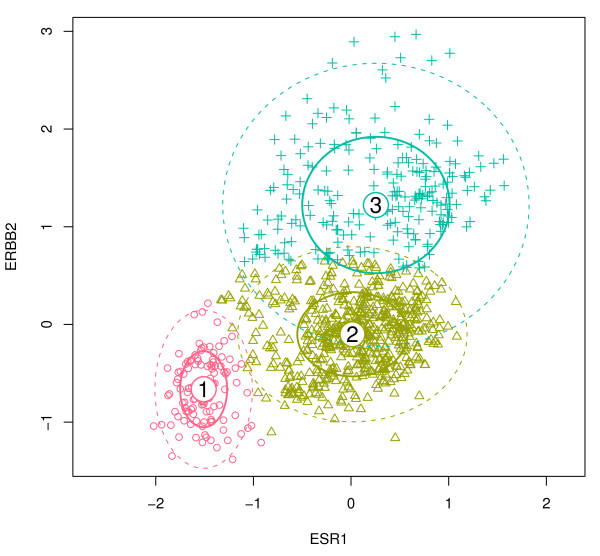
**ER/HER2 subtypes**. Expression of ESR1 (ER) versus ERBB2 (HER2) for the combined dataset. A mixture of three Gaussians is fitted to the data. Clusters 1, 2, and 3 represent the ER-/HER2-, ER+/HER2-, and HER2+ subtypes, respectively.

The triple-negative class, also called basal-like, is a group of breast cancer that are ER (also known as ESR1), PR (progesterone receptor), and HER2 (also known as ERBB2) [53]. We sought to reproduce the same biological results as previous reports which have identified subtype-specific signatures that predict metastasis [51,54]. We followed the procedure described by Desmedt et al. [51], and assessed their list of gene modules which are intended to represent different biological functions such as tumor invasion, immune response, angiogenesis, apoptosis, proliferation, and ER and HER2 signaling (see Additional File 1 for details). We clustered the samples based on their ER and HER2 module scores into three subgroups, ER-/HER2-, ER+/HER2-, and HER2+, shown in Figure 7 and Table 3.

**Table 3:** Breakdown of samples for each cancer subtype

	Class	< 5 years	≥ 5 years	Total
1	ER-/HER2-	35	80	115
2	ER+/HER2-	107	423	530
3	HER2+	55	164	219

We reran our analysis, consisting of training the centroid classifier on the MSigDB set statistics, on each subgroup. Table 4 shows the top gene sets for each subgroup for the set centroid statistic (see Additional File 1 for others statistics). The set centroid, set medoid, and set median show enrichment for genes from the AURKA module in the ER+/HER2- as expected, and to a lesser extent an immune response signature (STAT1 module) in ER-/HER2-, manifesting as IFN-*γ*-related sets in Table 4. Only in the ER-/HER- subgroup did these set statistics result in substantially different top gene sets. We also plotted the Kolmogorov-Smirnov statistics for how enriched were all 5414 MSigDB sets in genes from each of the modules from [51] (Additional File 1, Figure 11), showing that in contrast to the other set statistics, the set PC, set *t*-statistic and to some extent the set U statistic, exhibit more pronounced enrichment of Desmedt's module genes at the top and bottom of the sorted set list, indicating that the sets with large weight, either positive or negative, contain more of the genes defined in Desmedt's modules, and suggesting the same underlying biology as the modules.

**Table 4:** Top gene sets for each ER/HER2 subtype

Class	#	MSigDB Set	Cat.	Description	Sign
ER-/HER2-	1	chr7q12	Cl	Genes in cytogenetic band chr7q12	1
	2	COLLER_MYC_DN	C2	Genes down-regulated by MYC in 293T (transformed fetal renal cell).	-1
	3	IFNGPATHWAY	C2	IFN gamma signaling pathway	1
	4	GRANDVAUX_IFN_NOT_IRF3_UP	C2	Genes up-regulated by interferon-alpha, beta but not by IRF3 in Jurkat (T cell)	1
	5	GNF2_ST13	C4	Neighborhood of ST13	-1
	6	GNF2_CD48	C4	Neighborhood of CD48	1
	7	GNF2_GLTSCR2	C4	Neighborhood of GLTSCR2	-1
	8	MENSE_HYPOXIA_DN	C2	List of Hypoxia-suppressed genes found in both Astrocytes and HeLa Cells	-1
	9	HSA03010_RIBOSOME	C2	Genes involved in ribosome	-1
	10	GCM_TPT1	C4	Neighborhood of TPT1	-1

ER+/HER2-	1	GNF2_MKI67	C4	Neighborhood of MKI67	-1
	2	GNF2_TTK	C4	Neighborhood of TTK	-1
	3	GNF2_HMMR	C4	Neighborhood of HMMR	-1
	4	GNF2_CCNA2	C4	Neighborhood of CCNA2	-1
	5	GNF2_SMC2L1	C4	Neighborhood of SMC2L1	-1
	6	GNF2_ESPL1	C4	Neighborhood of ESPL1	-1
	7	GNF2_CDC20	C4	Neighborhood of CDC20	-1
	8	GNF2_H2AFX	C4	Neighborhood of H2AFX	-1
	9	GNF2_RRM2	C4	Neighborhood of RRM2	-1
	10	ZHAN_MM_CD138_PR_VS_ REST	C2	50 top ranked SAM-defined over-expressed genes in each subgroup_PR	-1

HER2+	1	chr4p	Cl	Genes in cytogenetic band chr4p	-1
	2	chrlqll	Cl	Genes in cytogenetic band chrlqll	1
	3	DAC_FIBRO_DN	C2	Downregulated by DAC treatment in LD419 fibroblast cells	-1
	4	GNF2_MKI67	C4	Neighborhood of MKI67	-1
	5	GNF2_CCNA2	C4	Neighborhood of CCNA2	-1
	6	GNF2_TTK	C4	Neighborhood of TTK	-1
	7	GNF2_H2AFX	C4	Neighborhood of H2AFX	-1
	8	GNF2_HMMR	C4	Neighborhood of HMMR	-1
	9	CROONQUIST_L6_RAS_DN	C2	Genes dowmregulated in multiple myeloma cells exposed to the pro-proliferative cytokine IL-6 versus those with N-ras-activating mutations.	-1
	10	CROONQUIST_L6_STARVE_UP	C2	Genes upregulated in multiple myeloma cells exposed to the pro-proliferative cytokine IL-6 versus those that were IL-6-starved.	-1

Top 10 MSigDB sets for ER/HER2 molecular subtypes, chosen by the centroid classifier using the set centroid statistic. Sign = -1 if expression is negatively associated with long-term survival, and vice versa.

### Do the Gene Sets Point to the Same Biology As the Genes?

In this section we investigate whether the top gene sets reflect the same underlying biology as the top genes. In the combined data, we trained three types of classifier: ℓ_2_-penalised logistic regression (R package penalized[55]), SVM with linear kernel (R package kernlab[56]), and the centroid classifier. Each classifier was trained on the genes and the gene set statistics (set centroids, see Additional File 1 for others), for a total of six models.

For each model, we ranked the features by the absolute value of their weights. We then selected the top 512 genes, which is a high enough number of genes producing a high AUC after which AUC does not increase much, and is a much higher number of genes than many published metastatic signatures. Other cutoffs (256, 1024, 2048) exhibited similar results (not shown). For each of the top ranked sets, we then checked how many of the top ranked genes belonged to that set, using the same classifier (i.e., centroid genes to centroid sets, logistic regression genes to logistic regression sets, SVM genes to SVM sets). The number of genes belonging to each set was quantified using a one-sided Fisher exact test.

As shown in Table 5, there is significant overlap between the top sets and top genes found by the centroid classifier. In comparison, both logistic regression and the SVM show very little overlap. In other words, the top sets ranked by the centroid classifier, using the set centroid statistic, are over-represented in the top genes selected by the centroid classifier, indicating the same underlying biological processes associated with metastasis.

**Table 5:** Overlap between top genes and gene sets for different classifiers

Classifier	#	MSigDB set	p-value	matches	set size
CC	1	GNF2_MKI67	< l.00 × l0^-40^	31	47
	2	GNF2_TTK	< l.00 × l0^-40^	29	57
	3	GNF2_CCNA2	< l.00 × 10^-40^	48	99
	4	GNF2_HMMR	< 1.00 × 10^-40^	42	78
	5	GNF2_SMC2L1	< 1.00 × 10^-40^	26	51
	6	GNF2_CDC20	< 1.00 × 10^-40^	46	91
	7	GNF2_ESPL1	< 1.00 × 10^-40^	27	58
	8	GNF2_H2AFX	< 1.00 × 10^-40^	24	54
	9	GNF2_RRM2	< 1.00 × 10^-40^	32	68
	10	chrlqll	2.32 × 10^-6^	2	4

SVM	1	chr7q12	6.23 × 10^4^	1	1
	2	chr3qll	1.00	0	8
	3	chrxq	1.00	0	2
	4	BYSTRYKH_RUNX1_TARGETS_GLO-CUS	8.06 × 10^-3^	1	13
	5	TESTIS_EXPRESSED _GENES	7.28 × 10^-7^	4	107
	6	chr22q	1.00	0	6
	7	REGULATION_OF_G_PROTEIN_COU-PLED_RECEPTOR_PROTEIN_SIGNAL-ING_PATHWAY	4.28 × 10^-4^	2	48
	8	chr11p14	1.00	0	20
	9	TERCPATHWAY	1.00	0	15
	10	chrlq41	2.02 × 10^-4^	2	33

LR	1	chrSqll	1.00	0	8
	2	chr22q	1.00	0	6
	3	TERCPATHWAY	1.00	0	15
	4	chrxq	1.00	0	2
	5	BYSTRYKH_RUNX1_TARGETS_GLO-CUS	8.06 × 10^-3^	1	13
	6	HSA00130_UBIQUINONE_BIOSYNTHE-SIS	1.00	0	8
	7	chr20p	1.00	0	2
	8	chrlq41	1.29 × 10^-6^	3	33
	9	chr3q12	1.00	0	23
	10	BETA_TUBULIN_BINDING	1.00	0	12

Top 10 sets using the set centroid statistic using different classifiers, and the *p*-value for the number of top genes belonging to each of them (Fisher's exact test, one sided). CC is centroid classifier, LR is logistic regression.

## Conclusions

We have shown that classifiers based on sets, rather than individual genes, have equivalent predictive power but are more stable, and as a result may facilitate increased understanding of the biological mechanism relating to breast cancer prognosis. The likely explanation is that the expression of any given gene is a function of both its contextual regulation -- regulation under varying conditions both observed and unobserved (such as noisy transcription) -- as well as inherent variability due to germ-line variations and differences in host-tumour response between individuals [57]. The former variability can be used for prognostic purposes. However, the latter reduces the prognostic accuracy since patient-level variability is typically not considered when building prognostic models.

Furthermore, the C4 sets tended to produce better classifiers than the other MSigDB categories. This difference may be due to the fact that C4 sets are based on datasets with a large number of samples; however, other factors cannot be ruled out. Our results suggest that there is prognostic value in large-scale systematic efforts to compile sets of coexpressed genes [10,58].

Importantly, our results are in agreement with current understanding of the drivers of metastasis in breast cancer -- proliferation for ER+/HER2-, immune response for ER-/HER2-, and tumour invasion and immune response for HER2+ [51] -- suggesting our approach could be a useful one. Apart from patient prognosis, there is also potential for understanding the biological mechanisms responsible for response and resistance to anti-cancer therapies.

We have used simple set statistics to represent gene set activity. These statistics are computationally tractable and depend on predefined set memberships. Some set statistics are not always sensible; for example, the average expression of a gene set of may not be meaningful when the genes are negatively correlated or uncorrelated; different statistics may be optimal for different gene sets. Moreover, these statistics ignore the structure and dynamics of the genetic networks, which could be important in deciphering causal relationships between genes and phenotypes. However, reliable information about the detailed structure of human genetic networks is currently limited.

## Abbreviations

PCA: Principal Component Analysis; SVD: Singular Value Decomposition; SVM: Support Vector Machine; MSigDB: Molecular Signatures Database; AUC: Area Under Receiver Operating Characteristic Curve.

## Availability and requirements

Project name: Gene Set Statistics

Project homepage: http://www.csse.unimelb.edu.au/~gabraham/GSS

Operating system(s): Unix-like

Programming languages: R

License: GPL v3

Any restrictions for use by non-academics: none

## Authors' contributions

GA developed and implemented the methodology, and analysed the data. AK, IH, and JZ participated in developing the methodology and the analysis of the data. SL participated in the analysis of the data. All authors contributed to the preparation of the manuscript, and read and approved the final manuscript.

## Supplementary Material

Additional file 1 **supplementary**. Further details on data preprocessing, methodology, and results including internal validation and comparisons of the centroid classifier with other classifiers. Click here for file
