# Supplementary material for: Prediction of breast cancer prognosis using gene set statistics provides signature stability and biological context
Source: BMC Bioinformatics. 2010 May 25;11:277. doi: 10.1186/1471-2105-11-277 (PMC2895626; doi:10.1186/1471-2105-11-277)
Supplement: Additional file 1 — supplementary. Further details on data preprocessing, methodology, and results including internal validation and comparisons of the centroid classifier with other classifiers. [file 1471-2105-11-277-S1.PDF]

Prediction of breast cancer prognosis using gene set statistics provides signature stability and biological context — Supplementary Information

Gad Abraham, Adam Kowalczyk, Sherene Loi, Izhak Haviv, and Justin Zobel

May 20, 2010

Here we provide more details on issues not covered in the main manuscript.

1 Data

1.1 Mapping Probesets to Genes

For some genes, Affymetrix arrays have multiple probesets, due to design issues or to alternative splicing. Treating all splice variants of one gene identically may not make sense, since different protein isoforms may have radically different roles in the cell [1]. Therefore, we considered each gene's probesets separately (as if they were distinct genes). In this paper we will use the terms "genes" and "probesets" interchangeably unless the distinction is important.

1.2 Data Normalisation

We normalised the GSE2034 dataset using quantile normalisation implemented in the R [2] 2.10.1 environment, using Bioconductor [3] 2.5 packages `affy` [4] and `preprocessCore` [5]. GSE4922 was normalised by the original authors, using the global mean method. For GSE6532, we used the data supplied by the original authors, who normalised it using RMA. GSE7390 and GSE11121 were normalised by their respective authors using a scaling method. All expression levels were $\log_2$ transformed.

1.3 MSigDB Gene Sets

We used the Bioconductor package `GSEABase` [6] to parse the MSigDB XML files, and the package `hgu133a.db` [7] to map gene symbols to Affymetrix probesets. Of the 5452 sets, 5414 sets contained genes that could be mapped to the Affymetrix HG-U133A probesets. In total, the MSigDB sets cover 19782 unique probesets on each microarray. Note that the MSigDB sets already contains the van 't Veer breast cancer metastasis signature [8], as well as 210 KEGG pathways. The `GO.db` package [9] was used to map GO IDs to GO terms.

2 Methods

2.1 Classifiers

In addition to the centroid classifier, we tested the shrunken centroid [10] in the R package `pamr` [11], our implementation of the classifier from [8], and a support vector machine with a linear kernel (`kernlab` package [12]). We optimised the shrunken centroid's threshold and the SVM's number of features and its ℓ_2 penalty using nested random splits, where the data was randomly split into three parts: training, validation, and testing. The model was fit to the training

data, and its AUC calculated for its prediction on the validation data. This was repeated over a grid of values appropriate for each model type. The optimal hyperparameters were then chosen as the ones maximising the AUC over the validation set. The model was then refit using the optimal hyperparameters on the training and validation data together, and tested on the remaining test data. Its AUC over the test data is reported. The whole procedure is repeated B times, producing B classifiers (for each classifier type), with different sets of optimal hyperparameters. The procedure is performed separately for each of the five datasets.

2.1.1 van 't Veer et al. [8] classifier

There are conflicting descriptions of the exact form of the classifier used in [8]. In the original paper, it seems that the classifier classifies each sample using its Pearson correlation with each of the centroids of the positive and negative metastasis classes:

$$\hat{y}_i = \underset{j \in \{-1,1\}}{\operatorname{argmin}} \{\operatorname{Corr}(x_i, c_j)\}, \quad (1)$$

where $\operatorname{Corr}(\cdot)$ is the Pearson correlation, x_i is the ith sample of p genes, and c_j is the centroid of the j class where $j \in \{-1, 1\}$. In other publications [13, 14], the classifier said to be based on the the correlation of the sample with the positive class only, and a threshold on that correlation is used to determine which class is predicted:

$$\hat{y}_i = \begin{cases} 1 & \text{if} \quad \operatorname{Corr}(x_i, c_1) \geq \tau; \\ -1 & \text{otherwise}, \end{cases} \quad (2)$$

where c_1 is the centroid for the positive class, and τ is a user-specified threshold.

We implemented both approaches, denoting them here VV1 and VV2 respectively. For the VV1 approach, we did not choose a threshold but used the correlation with the positive class as the prediction.

The VV2 approach is identical to the centroid classifier used in our work when the samples have been normalised so that they have unit norm [15, pp. 202–203].

2.2 Bagging

The B classifiers are then combined into an ensemble classifiers, where for each new sample, all classifiers in the ensemble predict it and the final prediction is the average over these predictions. The predictions are kept in the form of a weight vector or probability (rather than just the predicted class) in order to produce smoother ROC curves and smoother AUC calculations. Each ensemble classifier is then used for the external validation.

In the case where it does not make sense to average a model's weights (for example, an SVM's weights are typically in dual form, i.e., for the samples and not the features), we averaged over the output of the classifiers and took that as the ensemble prediction. In this case, output was kept as a real number rather than discretised into classes, in order to reduce the variance of the AUC.

2.3 Discrimination

We measure discrimination using the Area Under the ROC Curve (AUC or AROC) [16], defined as

$$\widehat{\mathrm{AUC}} = \frac{1}{Y^+ Y^-} \sum_{i=1}^{Y^+} \sum_{j=1}^{Y^-} \left[I(y_i^+ > y_j^-) + \frac{1}{2} I(y_i^+ = y_j^-) \right], \quad (3)$$

where Y^+ and Y^- are the number of true positive and true negative labels, respectively; y^+ and y^- are the predictions for the true positive and true negative labels, respectively; and $I(\cdot)$ is the indicator function, $I(x) = 1$ when x is true and 0 otherwise. The sample AUC has the probabilistic interpretation as the (estimated) probability of correctly ranking two randomly chosen samples in

the correct order (i.e., short-term survival before long-term survival). AUC = 0.5 is equivalent to random ranking. Unlike the error rate (or the accuracy), it does not depend on the class balance of the dataset.

2.4 Set Statistics

2.4.1 Principal Component

One possible problem with PCA is *axis reflection* [17]. Since the eigenvalues of the covariance matrix are determined only up to a constant, different numerical implementations of PCA may result in eigenvectors of opposite signs. Furthermore, even bootstrap replications of the same data may yield flipped signs. This effect is increased in the presence of noise. When used as features for classification or regression, flipped signs result in flipped estimates of the parameters. Since there are usually differences between datasets, axis reflection especially manifests itself in negative correlation between the eigenvectors derived from each dataset. (Eigenvectors from different datasets may point in opposite directions since the gene set may change the sign of its correlation with the phenotype under different experimental conditions; we assume this is not the case.)

To mitigate the effects of axis reflection, the sign of the eigenvectors must be (arbitrarily) fixed. Since we are interested only in the first principal component, we must fix the sign of the first eigenvalue. To do this, we examine the sign of the inner product of the eigenvalue with one of the axes and flip the eigenvector's sign to get a fixed eigenvector v'_{ij}

$$v'_{1j} = \begin{cases} -v_{1j}, & \text{sign}(\langle v_{1j}, g \rangle) < 0, \\ v_{1j}, & \text{otherwise.} \end{cases} \tag{4}$$

where g is a vector $(0, 1, 1, \ldots, 1)^T$ of same length as v_{ij}. Note that, even after this correction, axis reflections might still occur, especially for eigenvectors that are almost exactly aligned with the chosen axis (angle close to zero), since the noise may perturb the angle slightly about zero, thereby arbitrarily flipping the eigenvector's sign. A second caveat with PCA is that although it finds the principal component that explains the most variance in the predictor variables, this principal component may or may not explain the variance in the response variable. A third and final caveat with PCA is that although it is intended to reduce the effects of noise on the data, it can itself be sensitive to noise and outliers. For example, while most of the data may lie along one direction (suggesting this direction is a good principal component), adding a few large outliers orthogonal to this direction may result in a different (orthogonal) principal component being chosen.

Other PCA variants have been proposed, for example, smoothed or penalized principal components [18, Ch. 9], and supervised PCA [19]. We have not implemented these here, since standard PCA is more common in the literature and the more sophisticated methods require further tuning.

2.5 Gene Ontology

GO enrichment was computed using `fisher.test` in R. Affymetrix probesets were mapped to GO terms using the Bioconductor package `GO.db` [9]. IEA terms (automatically assigned annotations) were not considered for the GO enrichment test.

2.6 MSigDB Sets

2.6.1 Enrichment for MSigDB Categories

Gene Set Enrichment Analysis (GSEA) [20, 21] uses the the counting formulation of the two-sided two-sample Kolmogorov-Smirnov statistic [22, p. 182], to quantify how genes belonging to some gene set are distributed relative to genes not in this set. Note that this statistic does not quantify the deviation from uniform randomness (which would require the one-sample Kolmogorov-Smirnov test), but deviation of sets from *each other*. Equivalently, we use the two-sided two-sample Kolmogorov-Smirnov statistic for testing for enrichment of categories belonging to a given MSigDB category (C1, C2, C3, C4, C5).

First we define the form of the Kolmogorov-Smirnov statistic we use here. Let $F(t)$ and $G(t)$ be the cdfs of the two continuous random variables X and Y. The null and alternative hypotheses are, respectively,

$$H_0 : F(t) = G(t) \text{ for all } t, \qquad H_A : F(t) \neq G(t) \text{ for at least one } t. \tag{5}$$

The two-sided two-sample Kolmogorov-Smirnov statistic is

$$K = \sup_t |F_n(t) - G_m(t)|, \tag{6}$$

where $F_n(t) = \frac{1}{n}\sum_{i=1}^{n} I(x_i \leq t)$ and $G_m(t) = \frac{1}{m}\sum_{i=1}^{m} I(y_i \leq t)$ are the empirical cdfs of the two samples (n samples from X and m samples from Y), respectively, and $I(\cdot)$ is the indicator function (1 if true and 0 otherwise). K is computed as

$$K = \max_{i=1,\dots,N} |F_n(z_i) - G_m(z_i)|, \tag{7}$$

where z are the combined samples $(x_1, \dots, x_n, y_1, \dots, y_m)$, that have been ordered in ascending order, such that $z_1 \leq z_2 \leq \cdots \leq z_N$, $N = m + n$. (Our formulation here differs from [22, pp. 178–179] in that we do not multiply K by $\frac{mn}{d}$.)

Under the assumption that X and Y are continuous random variables, there are no ties between $F_n(t)$ and $G_m(t)$, therefore at each t, the difference $F_n(t) - G_m(t)$ can either increase by $1/n$ or decrease by $1/m$, but not both. Hence, K can also be calculated using a cumulative sum S

$$K = \max_{i=1,\dots,N} |S|, \tag{8}$$

where

$$S_j = S_{j-1} + \delta_j, \quad \delta_j = \begin{cases} 1/n & \text{if } z_j \text{ is from } X, \\ -1/m & \text{if } z_j \text{ is from } Y. \end{cases} \quad j = 1, \dots, N, \tag{9}$$

and $S_0 = 0$.

In GSEA, the cumulative sum S is plotted to show the relative location of each gene set. Similarly, we plot S to show the location of the MSigDB categories in the ranked sets — for each category C_k, $k \in \{1, 2, 3, 4, 5\}$, we take X to represent the weights of the sets from C_k (weights are averages over the five datasets), and Y to represent the weights of the sets outside the category, i.e., $C_{\{1,2,3,4,5\}\setminus k}$. The cumulative sum S_k is then computed for each category C_k.

Kolmogorov-Smirnov p-values are conservative (larger) in the presence of ties [23, pp. 330–331], hence we do not correct for tied ranks.

2.7 Gene Modules

We used the gene modules defined by Desmedt et al. [24]. In our gene expression data, the modules were aggregated into one vector (the module score for each sample) using the weighted average of expression, where the weight is simply the sign of the Pearson correlation of the each gene with the module's prototype gene. The scores for each module were normalised to have zero median and inter-quartile range of one. Finally, using the module scores for the ERBB1 and ESR1 modules, we fit a three-component Gaussian mixture model with diagonal covariance (R package `flexmix` [25]), in order to find three distinct subgroups, ER−/HER2−, ER+/HER2−, and HER2+, as shown in Figure 7 and Table 3 in the main article.

3 Results

3.1 Internal Validation

Figures 1, 2, 3, 4, and 5, show results for internal validation of the centroid classifier, the SVM, the PAM classifier (shrunken centroid), and the two van 't Veer et al. [8] classifiers, respectively. For the centroid, recursive feature elimination (RFE) was used. For the other classifiers, all features (all genes or all gene sets) were used. The SVM and PAM classifiers were optimised as discussed in Section 2.1.

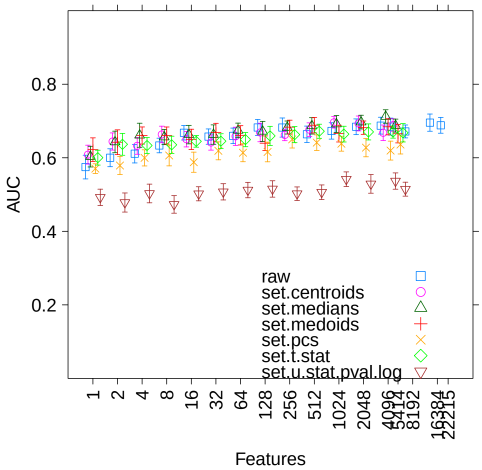

(a) GSE2034

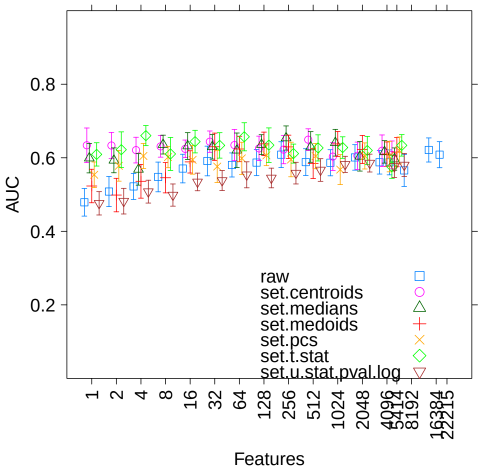

(b) GSE4922

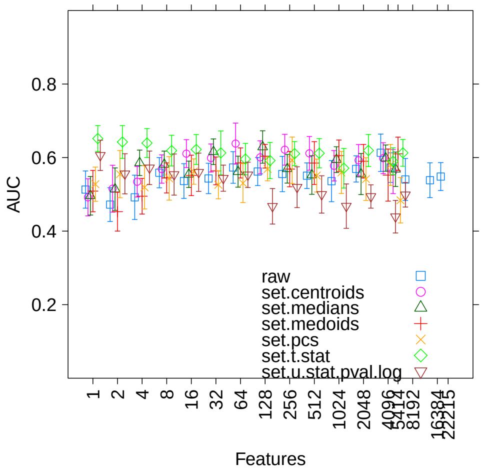

(c) GSE6532

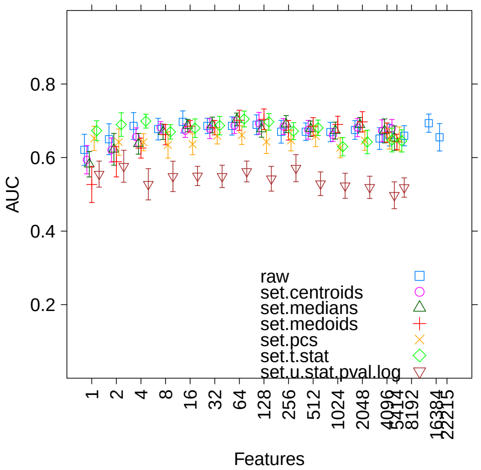

(d) GSE7390

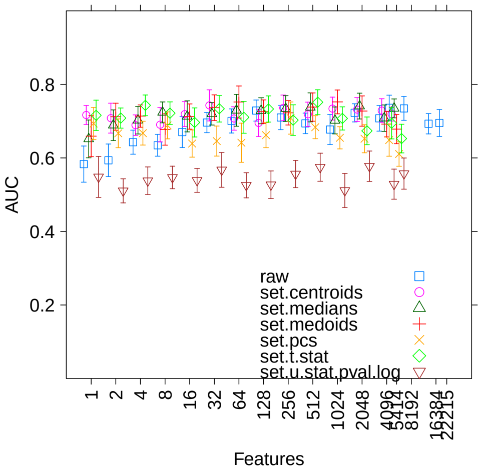

(e) GSE11121

Figure 1: Internal validation (mean and 95% CI for AUC) for centroid classifier with RFE

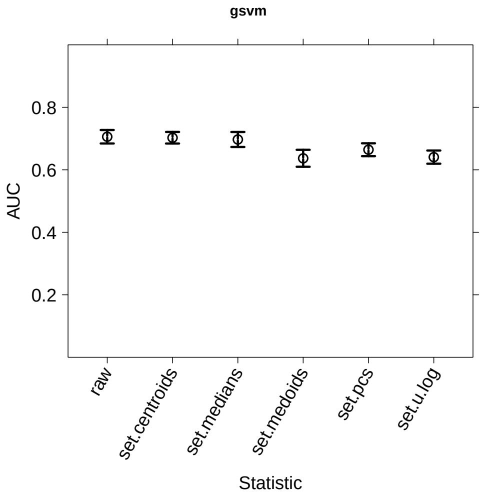

(a) GSE2034

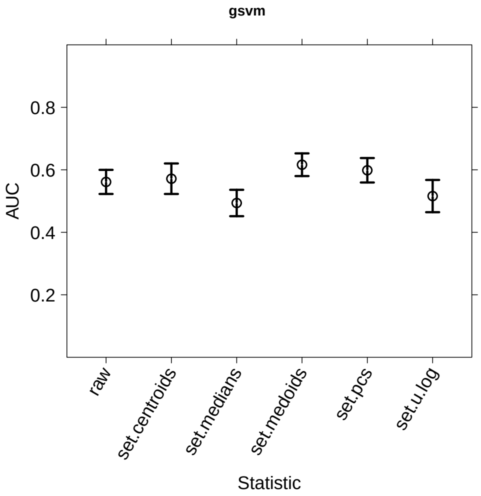

(b) GSE4922

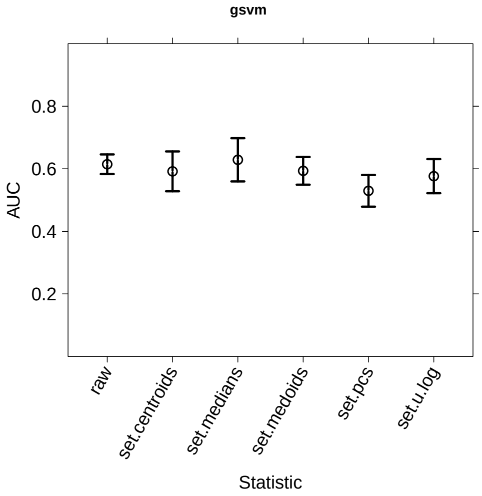

(c) GSE6532

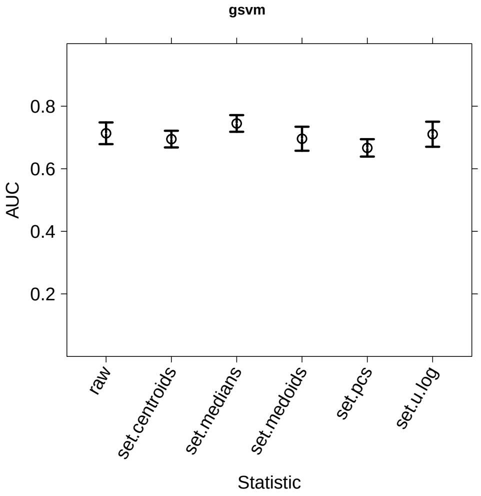

(d) GSE7390

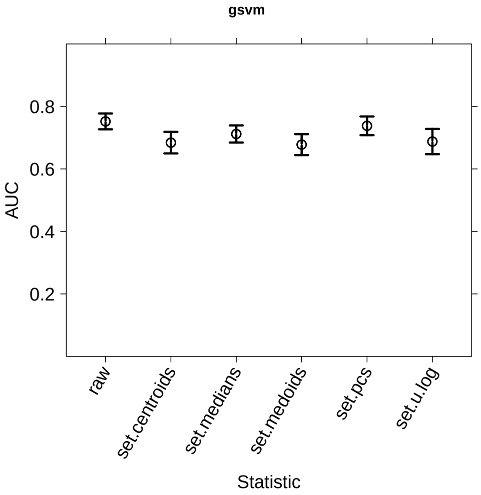

(e) GSE11121

Figure 2: Internal validation (mean and 95% CI for AUC) for SVM classifier, using all features

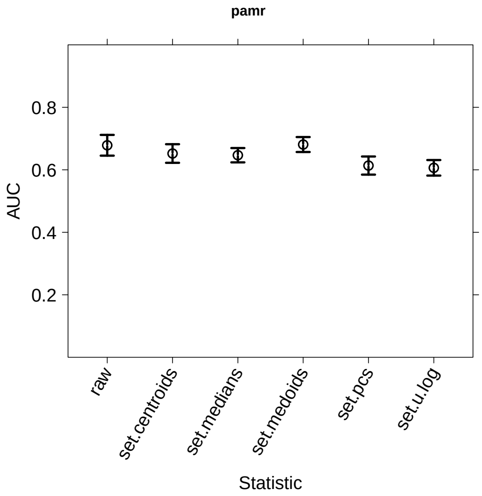

(a) GSE2034

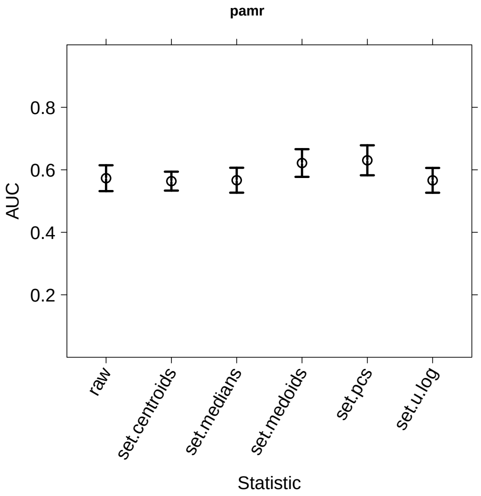

(b) GSE4922

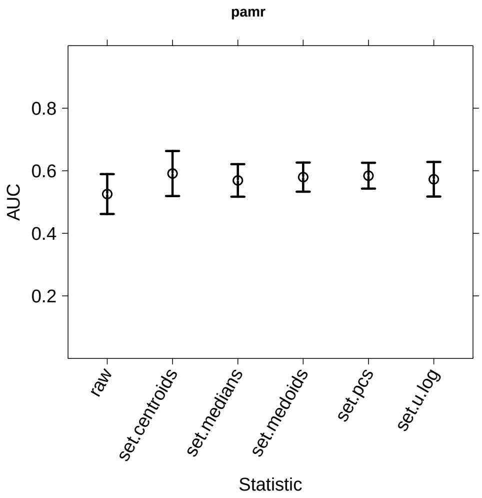

(c) GSE6532

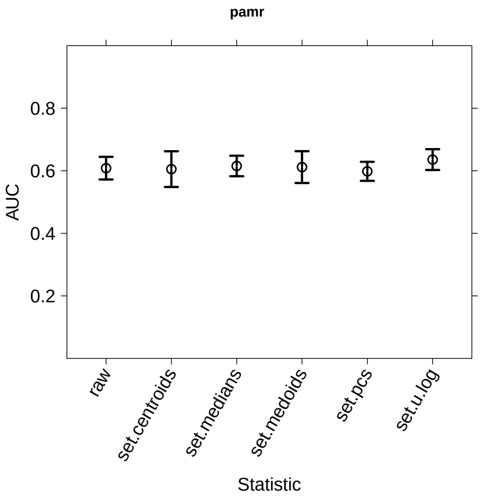

(d) GSE7390

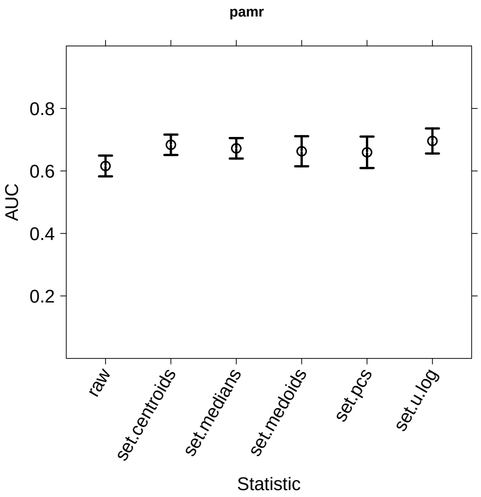

(e) GSE11121

Figure 3: Internal validation (mean and 95% CI for AUC) for PAM classifier

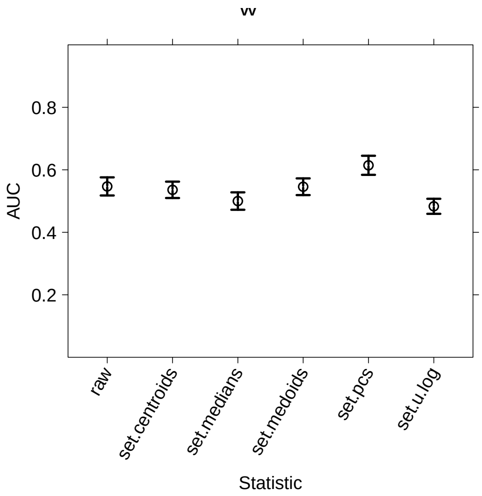

(a) GSE2034

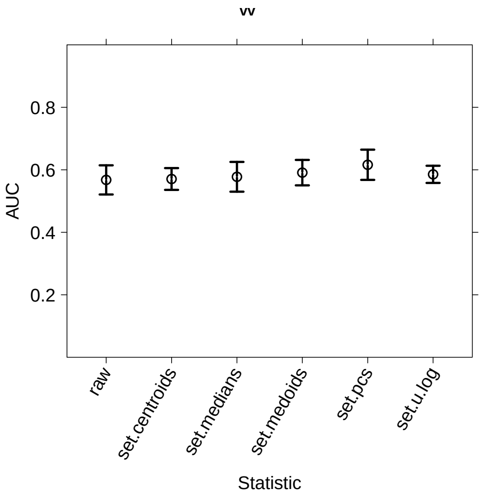

(b) GSE4922

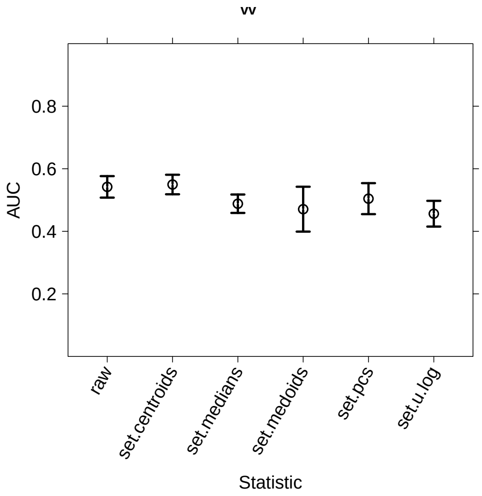

(c) GSE6532

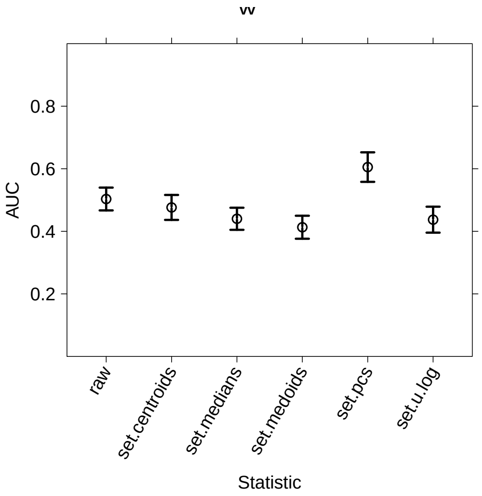

(d) GSE7390

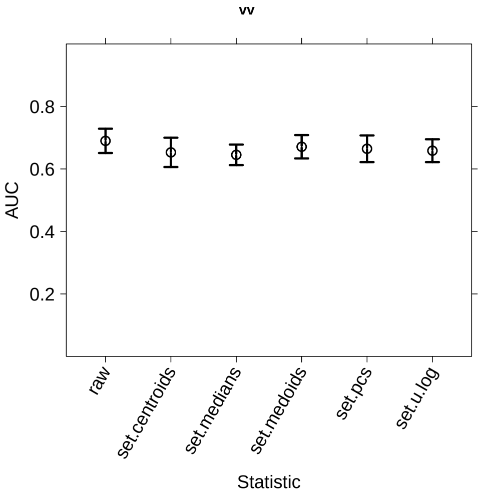

(e) GSE11121

Figure 4: Internal validation (mean and 95% CI for AUC) for VV1 classifier

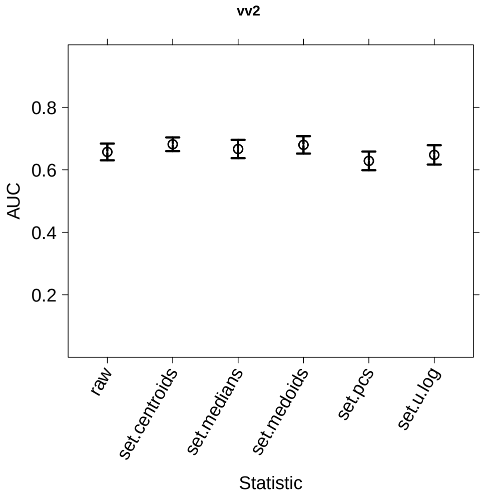

(a) GSE2034

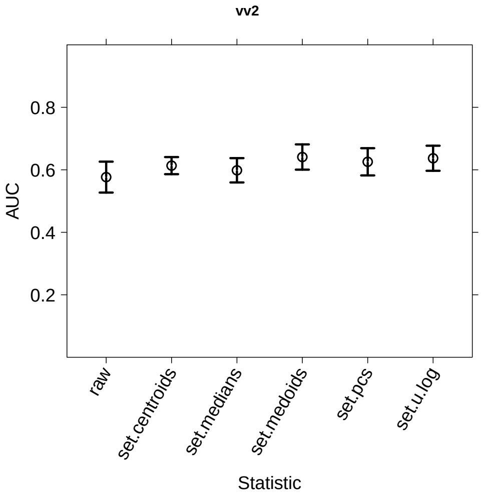

(b) GSE4922

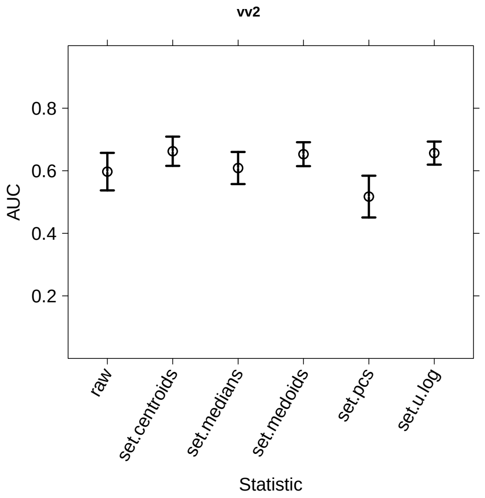

(c) GSE6532

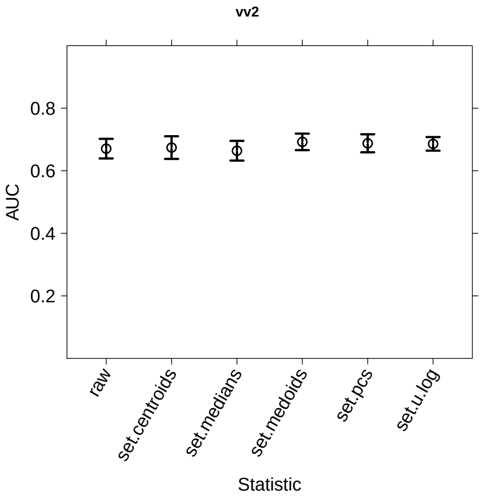

(d) GSE7390

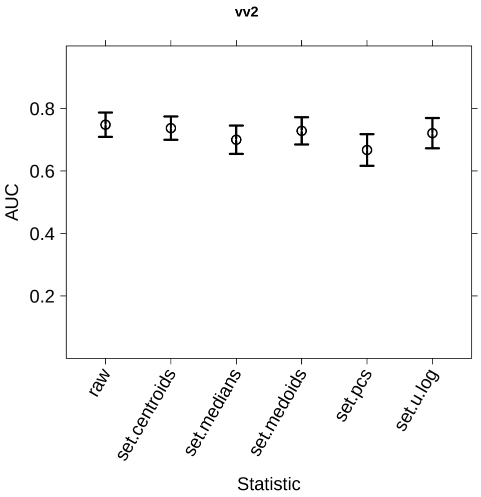

(e) GSE11121

Figure 5: Internal validation (mean and 95% CI for AUC) for VV2 classifier

3.2 External Validation

Figure 6 shows AUC for external validation for the different models.

3.2.1 Significance of AUC Differences

We used ANOVA to test for differences in AUC between the set statistics, produced by the centroid classifier. ANOVA is a reasonable since AUC is approximately normally distributed when it is not too close to zero or one. We present the results for 1, 8, 64, and 4096 features, as shown in Table 1. Taking into account multiple testing, the ANOVA shows that the AUC for set centroid, set median, and set t-statistic are not significantly different from the AUC for the individual genes. The AUC for set PC and set U statistic are significantly lower.

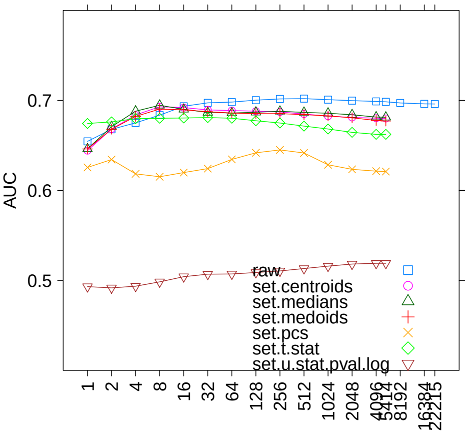

(a) Centroid with RFE (point estimate)

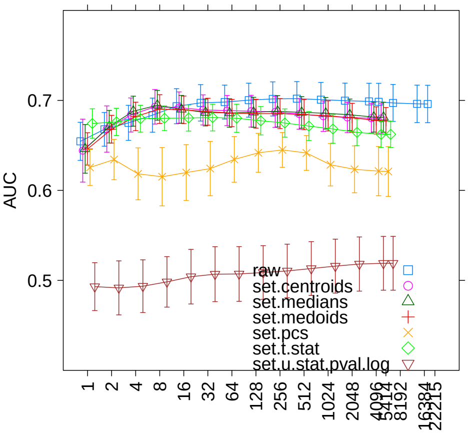

(b) Centroid with RFE (point + 95% confidence interval)

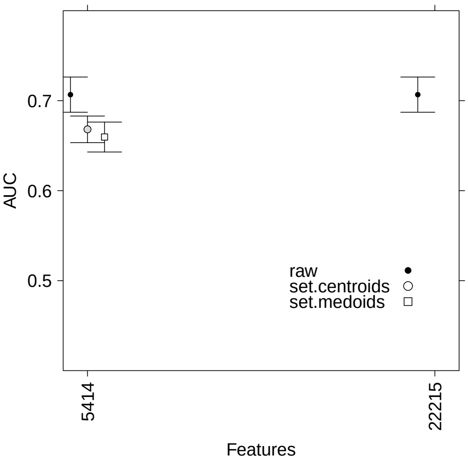

(c) SVM

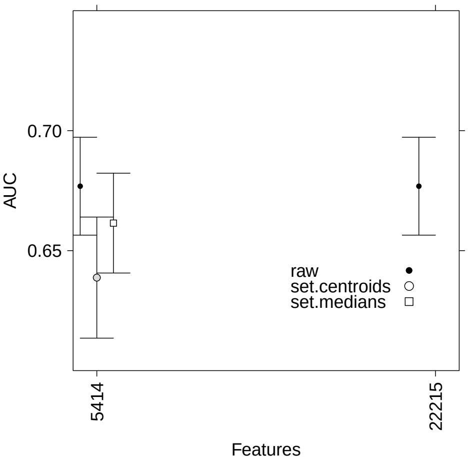

(d) PAM

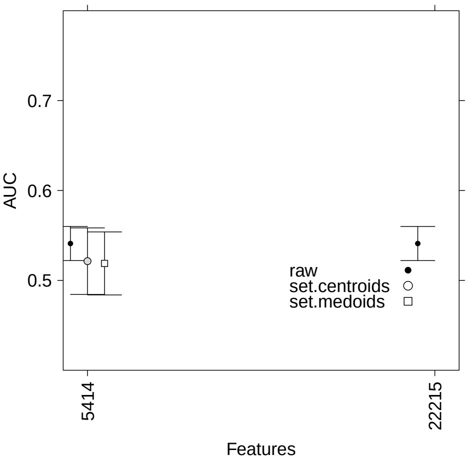

(e) VV1

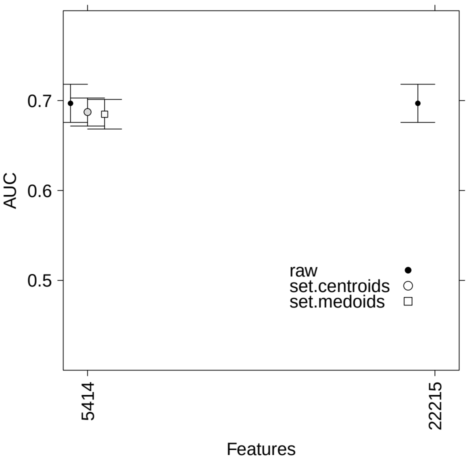

(f) VV2

Figure 6: External validation (mean and 95% CI for AUC) for all models

	Estimate	Std. Error	t value	Pr(>|t|)
(Intercept)	0.6545	0.0116	56.63	0.0000
set.centroids	-0.0103	0.0163	-0.63	0.5301
set.medians	-0.0083	0.0163	-0.51	0.6110
set.medoids	-0.0084	0.0163	-0.52	0.6073
set.pcs	-0.0290	0.0163	-1.77	0.0784
set.t.stat	0.0199	0.0163	1.22	0.2263
set.u.stat.pval.log	-0.1615	0.0163	-9.88	0.0000

(a) 1 feature/s

	Estimate	Std. Error	t value	Pr(>|t|)
(Intercept)	0.6836	0.0103	66.11	0.0000
set.centroids	0.0093	0.0146	0.64	0.5260
set.medians	0.0110	0.0146	0.75	0.4548
set.medoids	0.0067	0.0146	0.46	0.6452
set.pcs	-0.0684	0.0146	-4.68	0.0000
set.t.stat	-0.0035	0.0146	-0.24	0.8134
set.u.stat.pval.log	-0.1853	0.0146	-12.67	0.0000

(b) 8 feature/s

	Estimate	Std. Error	t value	Pr(>|t|)
(Intercept)	0.6982	0.0098	71.46	0.0000
set.centroids	-0.0095	0.0138	-0.68	0.4947
set.medians	-0.0121	0.0138	-0.88	0.3813
set.medoids	-0.0122	0.0138	-0.88	0.3781
set.pcs	-0.0637	0.0138	-4.61	0.0000
set.t.stat	-0.0180	0.0138	-1.30	0.1957
set.u.stat.pval.log	-0.1910	0.0138	-13.83	0.0000

(c) 64 feature/s

	Estimate	Std. Error	t value	Pr(>|t|)
(Intercept)	0.6989	0.0100	70.22	0.0000
set.centroids	-0.0187	0.0141	-1.33	0.1873
set.medians	-0.0174	0.0141	-1.24	0.2187
set.medoids	-0.0212	0.0141	-1.50	0.1353
set.pcs	-0.0775	0.0141	-5.51	0.0000
set.t.stat	-0.0366	0.0141	-2.60	0.0103
set.u.stat.pval.log	-0.1799	0.0141	-12.78	0.0000

(d) 4096 feature/s

Table 1: ANOVA of external-validation AUC different numbers of features. The AUC for individual genes is used as the intercept.

3.3 Top Gene Sets and GO Enrichment

Top 10 gene sets by average rank over the five datasets, using the set centroid statistic. GO enrichment p-values are from a Bonferroni-adjusted one-sided Fisher's exact test (30,330 tests). Sign=-1 if expression is negatively associated with long-term survival, and vice versa. The background list for the test includes all Affymetrix probesets that could be mapped to GO BP terms, excluding IEA annotations.

#	Set	Cat.	Sign	MSigDB Description	Enriched GO BP Terms (adj. p-value)
1	GNF2_MKI67	C4	-1	Neighborhood of MKI67	"phosphoinositide-mediated signaling": 1.95×10^{-10}, "spindle organization": 5.86×10^{-6}, "establishment of mitotic spindle localization": 1.10×10^{-5}, "kinetochore assembly": 5.48×10^{-5}, "mitotic chromosome condensation": 1.37×10^{-4}, "protein complex localization": 2.55×10^{-3}, "regulation of striated muscle development": 2.55×10^{-3}, "metaphase plate congression": 2.55×10^{-3}
2	GNF2_CCNA2	C4	-1	Neighborhood of CCNA2	"phosphoinositide-mediated signaling": $< 2.22\times10^{-16}$, "mitotic chromosome condensation": 4.35×10^{-14}, "DNA replication": 1.01×10^{-12}, "spindle organization": 1.37×10^{-9}, "establishment of mitotic spindle localization": 9.59×10^{-5}, "kinetochore assembly": 4.76×10^{-4}, "DNA repair": 5.78×10^{-3}, "mitosis": 9.44×10^{-3}
3	GNF2_TTK	C4	-1	Neighborhood of TTK	"phosphoinositide-mediated signaling": 4.05×10^{-16}, "DNA replication": 1.04×10^{-9}, "mitotic chromosome condensation": 1.32×10^{-8}, "regulation of striated muscle development": 3.76×10^{-3}, "metaphase plate congression": 3.76×10^{-3}
4	GNF2_HMMR	C4	-1	Neighborhood of HMMR	"phosphoinositide-mediated signaling": $< 2.22\times10^{-16}$, "mitotic cell cycle spindle assembly checkpoint": 1.26×10^{-11}, "spindle organization": 4.89×10^{-10}, "mitotic chromosome condensation": 8.46×10^{-8}, "cell proliferation": 6.22×10^{-6}, "DNA replication": 1.09×10^{-5}, "establishment of mitotic spindle localization": 5.33×10^{-5}, "kinetochore assembly": 2.65×10^{-4}, "protein complex localization": 8.29×10^{-3}, "regulation of striated muscle development": 8.29×10^{-3}, "metaphase plate congression": 8.29×10^{-3}
5	GNF2_CDC20	C4	-1	Neighborhood of CDC20	"phosphoinositide-mediated signaling": $< 2.22\times10^{-16}$, "spindle organization": 2.20×10^{-12}, "mitotic cell cycle spindle assembly checkpoint": 4.07×10^{-11}, "mitotic chromosome condensation": 1.52×10^{-9}, "cell proliferation": 8.96×10^{-9}, "mitosis": 1.83×10^{-8}, "establishment of mitotic spindle localization": 8.95×10^{-5}, "kinetochore assembly": 4.45×10^{-4}, "DNA replication": 7.83×10^{-3}
6	GNF2_SMC2L1	C4	-1	Neighborhood of SMC2L1	"mitotic cell cycle spindle assembly checkpoint": 5.15×10^{-13}, "mitotic chromosome condensation": 7.16×10^{-9}, "phosphoinositide-mediated signaling": 2.14×10^{-6}, "establishment of mitotic spindle localization": 1.31×10^{-5}, "kinetochore assembly": 6.51×10^{-5}, "protein complex localization": 2.90×10^{-3}, "DNA strand elongation during DNA replication": 2.90×10^{-3}, "regulation of striated muscle development": 2.90×10^{-3}, "metaphase plate congression": 2.90×10^{-3}, "cell proliferation": 2.94×10^{-3}, "nucleotide-excision repair, DNA gap filling": 3.56×10^{-3}
7	GNF2_H2AFX	C4	-1	Neighborhood of H2AFX	"phosphoinositide-mediated signaling": 5.38×10^{-11}, "kinetochore assembly": 3.12×10^{-5}, "mitotic chromosome condensation": 6.75×10^{-5}, "spindle organization": 7.76×10^{-4}, "protein complex localization": 1.67×10^{-3}, "regulation of striated muscle development": 1.67×10^{-3}, "metaphase plate congression": 1.67×10^{-3}

8	GNF2_ESPL1	C4	-1	Neighborhood of ESPL1	“cell proliferation”: 9.28×10^{-10}, “phosphoinositide-mediated signaling”: 5.54×10^{-7}, “mitosis”: 8.48×10^{-5}, “mitotic cell cycle spindle assembly checkpoint”: 1.33×10^{-4}, “protein complex localization”: 1.63×10^{-3}
9	GNF2_RRM2	C4	-1	Neighborhood of RRM2	“phosphoinositide-mediated signaling”: 4.52×10^{-15}, “mitotic cell cycle spindle assembly checkpoint”: 1.17×10^{-9}, “spindle organization”: 1.20×10^{-7}, “DNA replication”: 5.42×10^{-6}, “cell proliferation”: 1.97×10^{-5}, “establishment of mitotic spindle localization”: 4.09×10^{-5}, “kinetochore assembly”: 2.03×10^{-4}, “protein complex localization”: 6.80×10^{-3}, “regulation of striated muscle development”: 6.80×10^{-3}, “metaphase plate congression”: 6.80×10^{-3}
10	GNF2_PCNA	C4	-1	Neighborhood of PCNA	“phosphoinositide-mediated signaling”: $< 2.22\times10^{-16}$, “DNA replication”: 1.47×10^{-15}, “mitotic chromosome condensation”: 2.36×10^{-7}, “spindle organization”: 4.33×10^{-7}, “establishment of mitotic spindle localization”: 9.59×10^{-5}, “cell proliferation”: 4.18×10^{-4}, “DNA repair”: 4.33×10^{-4}, “kinetochore assembly”: 4.76×10^{-4}, “mitosis”: 9.44×10^{-3}

Table 2: set.centroids

#	Set	Cat.	Sign	MSigDB Description	Enriched GO BP Terms (adj. p-value)
1	GNF2_MKI67	C4	-1	Neighborhood of MKI67	"phosphoinositide-mediated signaling": 1.95×10^{-10}, "spindle organization": 5.86×10^{-6}, "establishment of mitotic spindle localization": 1.10×10^{-5}, "kinetochore assembly": 5.48×10^{-5}, "mitotic chromosome condensation": 1.37×10^{-4}, "protein complex localization": 2.55×10^{-3}, "regulation of striated muscle development": 2.55×10^{-3}, "metaphase plate congression": 2.55×10^{-3}
2	P21_P53_EARLY_DN	C2	-1	Down-regulated at early timepoints (4-8 hrs) following ectopic expression of p21 (CDKN1A) in OvCa cells, p53-dependent	"spindle organization": 4.64×10^{-5}, "phosphoinositide-mediated signaling": 9.80×10^{-3}
3	GNF2_HMMR	C4	-1	Neighborhood of HMMR	"phosphoinositide-mediated signaling": $< 2.22\times10^{-16}$, "mitotic cell cycle spindle assembly checkpoint": 1.26×10^{-11}, "spindle organization": 4.89×10^{-10}, "mitotic chromosome condensation": 8.46×10^{-8}, "cell proliferation": 6.22×10^{-6}, "DNA replication": 1.09×10^{-5}, "establishment of mitotic spindle localization": 5.33×10^{-5}, "kinetochore assembly": 2.65×10^{-4}, "protein complex localization": 8.29×10^{-3}, "regulation of striated muscle development": 8.29×10^{-3}, "metaphase plate congression": 8.29×10^{-3}
4	GNF2_TTK	C4	-1	Neighborhood of TTK	"phosphoinositide-mediated signaling": 4.05×10^{-16}, "DNA replication": 1.04×10^{-9}, "mitotic chromosome condensation": 1.32×10^{-8}, "regulation of striated muscle development": 3.76×10^{-3}, "metaphase plate congression": 3.76×10^{-3}
5	GNF2_CDC20	C4	-1	Neighborhood of CDC20	"phosphoinositide-mediated signaling": $< 2.22\times10^{-16}$, "spindle organization": 2.20×10^{-12}, "mitotic cell cycle spindle assembly checkpoint": 4.07×10^{-11}, "mitotic chromosome condensation": 1.52×10^{-9}, "cell proliferation": 8.96×10^{-9}, "mitosis": 1.83×10^{-8}, "establishment of mitotic spindle localization": 8.95×10^{-5}, "kinetochore assembly": 4.45×10^{-4}, "DNA replication": 7.83×10^{-3}
6	GNF2_CCNA2	C4	-1	Neighborhood of CCNA2	"phosphoinositide-mediated signaling": $< 2.22\times10^{-16}$, "mitotic chromosome condensation": 4.35×10^{-14}, "DNA replication": 1.01×10^{-12}, "spindle organization": 1.37×10^{-9}, "establishment of mitotic spindle localization": 9.59×10^{-5}, "kinetochore assembly": 4.76×10^{-4}, "DNA repair": 5.78×10^{-3}, "mitosis": 9.44×10^{-3}
7	GNF2_ESPL1	C4	-1	Neighborhood of ESPL1	"cell proliferation": 9.28×10^{-10}, "phosphoinositide-mediated signaling": 5.54×10^{-7}, "mitosis": 8.48×10^{-5}, "mitotic cell cycle spindle assembly checkpoint": 1.33×10^{-4}, "protein complex localization": 1.63×10^{-3}
8	GNF2_H2AFX	C4	-1	Neighborhood of H2AFX	"phosphoinositide-mediated signaling": 5.38×10^{-11}, "kinetochore assembly": 3.12×10^{-5}, "mitotic chromosome condensation": 6.75×10^{-5}, "spindle organization": 7.76×10^{-4}, "protein complex localization": 1.67×10^{-3}, "regulation of striated muscle development": 1.67×10^{-3}, "metaphase plate congression": 1.67×10^{-3}

9	GNF2_SMC2L1	C4	-1	Neighborhood of SMC2L1	“mitotic cell cycle spindle assembly checkpoint”: 5.15×10^{-13}, “mitotic chromosome condensation”: 7.16×10^{-9}, “phosphoinositide-mediated signaling”: 2.14×10^{-6}, “establishment of mitotic spindle localization”: 1.31×10^{-5}, “kinetochore assembly”: 6.51×10^{-5}, “protein complex localization”: 2.90×10^{-3}, “DNA strand elongation during DNA replication”: 2.90×10^{-3}, “regulation of striated muscle development”: 2.90×10^{-3}, “metaphase plate congression”: 2.90×10^{-3}, “cell proliferation”: 2.94×10^{-3}, “nucleotide-excision repair, DNA gap filling”: 3.56×10^{-3}
10	GNF2_RRM2	C4	-1	Neighborhood of RRM2	“phosphoinositide-mediated signaling”: 4.52×10^{-15}, “mitotic cell cycle spindle assembly checkpoint”: 1.17×10^{-9}, “spindle organization”: 1.20×10^{-7}, “DNA replication”: 5.42×10^{-6}, “cell proliferation”: 1.97×10^{-5}, “establishment of mitotic spindle localization”: 4.09×10^{-5}, “kinetochore assembly”: 2.03×10^{-4}, “protein complex localization”: 6.80×10^{-3}, “regulation of striated muscle development”: 6.80×10^{-3}, “metaphase plate congression”: 6.80×10^{-3}

Table 3: set.medians

#	Set	Cat.	Sign	MSigDB Description	Enriched GO BP Terms (adj. p-value)
1	GNF2_MKI67	C4	-1	Neighborhood of MKI67	"phosphoinositide-mediated signaling": 1.95×10^{-10}, "spindle organization": 5.86×10^{-6}, "establishment of mitotic spindle localization": 1.10×10^{-5}, "kinetochore assembly": 5.48×10^{-5}, "mitotic chromosome condensation": 1.37×10^{-4}, "protein complex localization": 2.55×10^{-3}, "regulation of striated muscle development": 2.55×10^{-3}, "metaphase plate congression": 2.55×10^{-3}
2	GNF2_TTK	C4	-1	Neighborhood of TTK	"phosphoinositide-mediated signaling": 4.05×10^{-16}, "DNA replication": 1.04×10^{-9}, "mitotic chromosome condensation": 1.32×10^{-8}, "regulation of striated muscle development": 3.76×10^{-3}, "metaphase plate congression": 3.76×10^{-3}
3	GNF2_CCNA2	C4	-1	Neighborhood of CCNA2	"phosphoinositide-mediated signaling": $< 2.22\times10^{-16}$, "mitotic chromosome condensation": 4.35×10^{-14}, "DNA replication": 1.01×10^{-12}, "spindle organization": 1.37×10^{-9}, "establishment of mitotic spindle localization": 9.59×10^{-5}, "kinetochore assembly": 4.76×10^{-4}, "DNA repair": 5.78×10^{-3}, "mitosis": 9.44×10^{-3}
4	GNF2_HMMR	C4	-1	Neighborhood of HMMR	"phosphoinositide-mediated signaling": $< 2.22\times10^{-16}$, "mitotic cell cycle spindle assembly checkpoint": 1.26×10^{-11}, "spindle organization": 4.89×10^{-10}, "mitotic chromosome condensation": 8.46×10^{-8}, "cell proliferation": 6.22×10^{-6}, "DNA replication": 1.09×10^{-5}, "establishment of mitotic spindle localization": 5.33×10^{-5}, "kinetochore assembly": 2.65×10^{-4}, "protein complex localization": 8.29×10^{-3}, "regulation of striated muscle development": 8.29×10^{-3}, "metaphase plate congression": 8.29×10^{-3}
5	GNF2_SMC2L1	C4	-1	Neighborhood of SMC2L1	"mitotic cell cycle spindle assembly checkpoint": 5.15×10^{-13}, "mitotic chromosome condensation": 7.16×10^{-9}, "phosphoinositide-mediated signaling": 2.14×10^{-6}, "establishment of mitotic spindle localization": 1.31×10^{-5}, "kinetochore assembly": 6.51×10^{-5}, "protein complex localization": 2.90×10^{-3}, "DNA strand elongation during DNA replication": 2.90×10^{-3}, "regulation of striated muscle development": 2.90×10^{-3}, "metaphase plate congression": 2.90×10^{-3}, "cell proliferation": 2.94×10^{-3}, "nucleotide-excision repair, DNA gap filling": 3.56×10^{-3}
6	GNF2_CDC20	C4	-1	Neighborhood of CDC20	"phosphoinositide-mediated signaling": $< 2.22\times10^{-16}$, "spindle organization": 2.20×10^{-12}, "mitotic cell cycle spindle assembly checkpoint": 4.07×10^{-11}, "mitotic chromosome condensation": 1.52×10^{-9}, "cell proliferation": 8.96×10^{-9}, "mitosis": 1.83×10^{-8}, "establishment of mitotic spindle localization": 8.95×10^{-5}, "kinetochore assembly": 4.45×10^{-4}, "DNA replication": 7.83×10^{-3}
7	GNF2_ESPL1	C4	-1	Neighborhood of ESPL1	"cell proliferation": 9.28×10^{-10}, "phosphoinositide-mediated signaling": 5.54×10^{-7}, "mitosis": 8.48×10^{-5}, "mitotic cell cycle spindle assembly checkpoint": 1.33×10^{-4}, "protein complex localization": 1.63×10^{-3}
8	GNF2_H2AFX	C4	-1	Neighborhood of H2AFX	"phosphoinositide-mediated signaling": 5.38×10^{-11}, "kinetochore assembly": 3.12×10^{-5}, "mitotic chromosome condensation": 6.75×10^{-5}, "spindle organization": 7.76×10^{-4}, "protein complex localization": 1.67×10^{-3}, "regulation of striated muscle development": 1.67×10^{-3}, "metaphase plate congression": 1.67×10^{-3}

9	GNF2_RRM2	C4	-1	Neighborhood of RRM2	“phosphoinositide-mediated signaling”: 4.52×10^{-15}, “mitotic cell cycle spindle assembly checkpoint”: 1.17×10^{-9}, “spindle organization”: 1.20×10^{-7}, “DNA replication”: 5.42×10^{-6}, “cell proliferation”: 1.97×10^{-5}, “establishment of mitotic spindle localization”: 4.09×10^{-5}, “kinetochore assembly”: 2.03×10^{-4}, “protein complex localization”: 6.80×10^{-3}, “regulation of striated muscle development”: 6.80×10^{-3}, “metaphase plate congression”: 6.80×10^{-3}
10	GNF2_PCNA	C4	-1	Neighborhood of PCNA	“phosphoinositide-mediated signaling”: $< 2.22 \times 10^{-16}$, “DNA replication”: 1.47×10^{-15}, “mitotic chromosome condensation”: 2.36×10^{-7}, “spindle organization”: 4.33×10^{-7}, “establishment of mitotic spindle localization”: 9.59×10^{-5}, “cell proliferation”: 4.18×10^{-4}, “DNA repair”: 4.33×10^{-4}, “kinetochore assembly”: 4.76×10^{-4}, “mitosis”: 9.44×10^{-3}

Table 4: set.medoids

#	Set	Cat.	Sign	MSigDB Description	Enriched GO BP Terms (adj. p-value)
1	STEMCELL_NEURAL_UP	C2	-1	Enriched in mouse neural stem cells, compared to differentiated brain and bone marrow cells	"anaphase-promoting complex-dependent proteasomal ubiquitin-dependent protein catabolic process": 2.78×10^{-12}, "RNA splicing": 3.15×10^{-9}, "negative regulation of ubiquitin-protein ligase activity during mitotic cell cycle": 4.33×10^{-9}, "positive regulation of ubiquitin-protein ligase activity during mitotic cell cycle": 2.00×10^{-8}, "DNA replication": 2.65×10^{-7}, "nucleosome assembly": 2.23×10^{-6}, "DNA repair": 7.43×10^{-6}, "translation": 1.30×10^{-5}, "mRNA processing": 3.42×10^{-5}, "cholesterol biosynthetic process": 2.36×10^{-4}, "response to UV-A": 1.07×10^{-3}, "nucleotide-excision repair, DNA gap filling": 2.00×10^{-3}, "regulation of translational initiation": 2.32×10^{-3}, "DNA damage checkpoint": 3.88×10^{-3}
2	module_54	C4	-1	Genes in module_54	"DNA replication": $< 2.22\times10^{-16}$, "regulation of cyclin-dependent protein kinase activity": $< 2.22\times10^{-16}$, "phosphoinositide-mediated signaling": 9.52×10^{-14}, "nucleotide-excision repair, DNA gap filling": 4.86×10^{-7}, "mitotic chromosome condensation": 6.06×10^{-7}, "DNA repair": 2.17×10^{-6}, "mitotic cell cycle spindle assembly checkpoint": 2.81×10^{-6}, "cell proliferation": 6.29×10^{-6}, "G1 phase of mitotic cell cycle": 1.08×10^{-4}, "regulation of mitosis": 3.22×10^{-4}, "spindle organization": 6.66×10^{-4}, "mitotic sister chromatid segregation": 3.50×10^{-3}
3	NUCLEUS	C5	-1	Genes annotated by the GO term GO:0005634. A membrane-bounded organelle of eukaryotic cells in which chromosomes are housed and replicated. In most cells, the nucleus contains all of the cell's chromosomes except the organellar chromosomes, and is the site of RNA synthesis and processing. In some species, or in specialized cell types, RNA metabolism or DNA replication may be absent.	"RNA splicing": $< 2.22\times10^{-16}$, "nuclear mRNA splicing, via spliceosome": $< 2.22\times10^{-16}$, "negative regulation of transcription, DNA-dependent": $< 2.22\times10^{-16}$, "transcription initiation from RNA polymerase II promoter": $< 2.22\times10^{-16}$, "positive regulation of transcription from RNA polymerase II promoter": 7.21×10^{-16}, "regulation of transcription from RNA polymerase II promoter": 1.33×10^{-15}, "androgen receptor signaling pathway": 7.35×10^{-15}, "response to DNA damage stimulus": 3.64×10^{-13}, "nucleosome assembly": 4.09×10^{-13}, "DNA repair": 3.07×10^{-10}, "positive regulation of transcription, DNA-dependent": 4.87×10^{-10}, "mRNA export from nucleus": 1.26×10^{-9}, "negative regulation of transcription": 2.83×10^{-9}, "RNA elongation from RNA polymerase II promoter": 2.78×10^{-8}, "DNA replication": 1.08×10^{-7}, "protein import into nucleus, translocation": 2.51×10^{-7}, "transcription, DNA-dependent": 4.70×10^{-7}, "transcription from RNA polymerase II promoter": 1.11×10^{-6}, "protein import into nucleus": 1.43×10^{-6}, "telomere maintenance": 2.23×10^{-6}, "nucleocytoplasmic transport": 2.54×10^{-6}, "spliceosomal snRNP biogenesis": 3.66×10^{-6}, "spliceosome assembly": 4.19×10^{-6}, "chromatin remodeling": 4.41×10^{-6}, "reciprocal meiotic recombination": 5.77×10^{-6}, "regulation of transcription, DNA-dependent": 9.66×10^{-6}, "DNA methylation": 1.06×10^{-5}, "negative regulation of transcription from RNA polymerase II promoter": 1.09×10^{-5}, "DNA recombination": 1.51×10^{-5}, "cell death": 3.99×10^{-5}, "DNA replication initiation": 1.27×10^{-4}, "nucleotide-excision repair, DNA damage removal": 1.72×10^{-4}, "negative regulation of DNA replication": 2.35×10^{-4}, "provirus integration": 2.44×10^{-4}, "mRNA processing": 1.08×10^{-3}, "double-strand break repair via nonhomologous end joining": 1.13×10^{-3}, "snRNA processing": 1.13×10^{-3}, "adrenal gland development": 1.13×10^{-3}, "metanephros development": 5.34×10^{-3}

4	STEMCELL_EMBRYONIC_UP	C2	-1	Enriched in mouse embryonic stem cells, compared to differentiated brain and bone marrow cells	"anaphase-promoting complex-dependent proteasomal ubiquitin-dependent protein catabolic process": 2.98×10^{-11}, "DNA replication": 8.15×10^{-9}, "negative regulation of ubiquitin-protein ligase activity during mitotic cell cycle": 4.36×10^{-8}, "RNA splicing": 1.07×10^{-7}, "positive regulation of ubiquitin-protein ligase activity during mitotic cell cycle": 1.23×10^{-7}, "regulation of cyclin-dependent protein kinase activity": 1.22×10^{-5}, "DNA damage checkpoint": 1.45×10^{-5}, "cell cycle arrest": 1.03×10^{-4}, "nucleosome assembly": 4.19×10^{-4}, "G1/S transition of mitotic cell cycle": 1.06×10^{-3}, "DNA repair": 1.47×10^{-3}, "primary microRNA processing": 6.54×10^{-3}
5	NUCLEOBASE__NUCLEOSIDE__NUCLEOTIDE_AND_NUCLEIC_ACID_METABOLIC_PROCESS	C5	1	Genes annotated by the GO term GO:0006139. The chemical reactions and pathways involving nucleobases, nucleosides, nucleotides and nucleic acids.	"regulation of transcription from RNA polymerase II promoter": $< 2.22\times10^{-16}$, "transcription from RNA polymerase II promoter": $< 2.22\times10^{-16}$, "negative regulation of transcription from RNA polymerase II promoter": $< 2.22\times10^{-16}$, "RNA splicing": $< 2.22\times10^{-16}$, "negative regulation of transcription": $< 2.22\times10^{-16}$, "positive regulation of transcription from RNA polymerase II promoter": $< 2.22\times10^{-16}$, "DNA repair": $< 2.22\times10^{-16}$, "DNA replication": $< 2.22\times10^{-16}$, "transcription initiation from RNA polymerase II promoter": $< 2.22\times10^{-16}$, "regulation of transcription, DNA-dependent": $< 2.22\times10^{-16}$, "nucleobase, nucleoside, nucleotide and nucleic acid metabolic process": $< 2.22\times10^{-16}$, "mRNA processing": $< 2.22\times10^{-16}$, "positive regulation of transcription, DNA-dependent": $< 2.22\times10^{-16}$, "RNA processing": $< 2.22\times10^{-16}$, "nuclear mRNA splicing, via spliceosome": $< 2.22\times10^{-16}$, "positive regulation of transcription": 2.85×10^{-14}, "RNA elongation from RNA polymerase II promoter": 5.25×10^{-14}, "negative regulation of transcription, DNA-dependent": 1.29×10^{-13}, "reciprocal meiotic recombination": 1.35×10^{-11}, "negative regulation of transcription factor activity": 6.59×10^{-11}, "positive regulation of NF-kappaB transcription factor activity": 2.44×10^{-10}, "DNA recombination": 6.93×10^{-10}, "negative regulation of DNA replication": 2.23×10^{-8}, "DNA damage checkpoint": 4.52×10^{-8}, "spliceosome assembly": 7.62×10^{-8}, "mRNA splice site selection": 9.69×10^{-8}, "androgen receptor signaling pathway": 1.96×10^{-7}, "nucleotide-excision repair, DNA damage removal": 4.02×10^{-7}, "DNA-dependent DNA replication": 4.22×10^{-7}, "transcription from RNA polymerase III promoter": 2.86×10^{-6}, "negative regulation of cell growth": 1.18×10^{-5}, "telomere maintenance via telomerase": 3.49×10^{-5}, "response to DNA damage stimulus": 1.17×10^{-4}, "nucleosome disassembly": 1.52×10^{-4}, "mRNA polyadenylation": 1.52×10^{-4}, "spliceosomal snRNP biogenesis": 2.18×10^{-4}, "telomere maintenance": 4.11×10^{-4}, "DNA fragmentation during apoptosis": 4.11×10^{-4}, "DNA replication initiation": 4.11×10^{-4}, "nucleotide-excision repair": 6.61×10^{-4}, "B cell lineage commitment": 6.61×10^{-4}, "DNA methylation": 1.67×10^{-3}, "transcription, DNA-dependent": 2.58×10^{-3}, "transcription": 2.64×10^{-3}, "double-strand break repair via nonhomologous end joining": 2.88×10^{-3}, "snRNA processing": 2.88×10^{-3}, "cell aging": 3.37×10^{-3}, "double-strand break repair": 3.37×10^{-3}, "positive regulation of specific transcription from RNA polymerase II promoter": 4.42×10^{-3}, "nucleosome assembly": 6.26×10^{-3}, "postreplication repair": 6.77×10^{-3}

6	module_98	C4	-1	Genes in module_98	"nucleotide-excision repair, DNA gap filling": $< 2.22\times10^{-16}$, "nuclear mRNA splicing, via spliceosome": $< 2.22\times10^{-16}$, "RNA splicing": $< 2.22\times10^{-16}$, "DNA repair": 3.04×10^{-10}, "DNA replication": 1.53×10^{-9}, "regulation of transcription from RNA polymerase II promoter": 1.35×10^{-7}, "protein import into nucleus, translocation": 4.69×10^{-7}, "transcription initiation from RNA polymerase II promoter": 8.29×10^{-7}, "RNA elongation from RNA polymerase II promoter": 4.98×10^{-6}, "mRNA splice site selection": 2.72×10^{-4}, "transcription from RNA polymerase II promoter": 3.00×10^{-4}, "NLS-bearing substrate import into nucleus": 3.37×10^{-3}, "regulation of cyclin-dependent protein kinase activity": 5.52×10^{-3}, "anaphase-promoting complex-dependent proteasomal ubiquitin-dependent protein catabolic process": 9.73×10^{-3}
7	TARTE_PLASMA_BLASTIC	C2	-1	Genes overexpressed in mature plasma cells isolated from tonsils (TPCs) and mature plasma cells isolated from bone marrow (BMPCs) as compared to polyclonal plasmablastic cells (PPCs).	"anaphase-promoting complex-dependent proteasomal ubiquitin-dependent protein catabolic process": 2.00×10^{-13}, "positive regulation of ubiquitin-protein ligase activity during mitotic cell cycle": 2.07×10^{-11}, "negative regulation of ubiquitin-protein ligase activity during mitotic cell cycle": 2.21×10^{-9}, "phosphoinositide-mediated signaling": 7.02×10^{-8}, "DNA repair": 6.77×10^{-4}, "mitotic metaphase/anaphase transition": 3.26×10^{-3}, "low-density lipoprotein particle clearance": 9.93×10^{-3}
8	module_198	C4	-1	Genes in module_198	"nucleotide-excision repair, DNA gap filling": $< 2.22\times10^{-16}$, "nuclear mRNA splicing, via spliceosome": $< 2.22\times10^{-16}$, "RNA splicing": 1.16×10^{-15}, "DNA repair": 5.49×10^{-11}, "DNA replication": 2.44×10^{-10}, "protein import into nucleus, translocation": 2.31×10^{-7}, "mRNA splice site selection": 1.64×10^{-5}, "nucleosome assembly": 1.03×10^{-3}, "transcription initiation from RNA polymerase II promoter": 1.41×10^{-3}, "regulation of transcription from RNA polymerase II promoter": 1.86×10^{-3}, "DNA recombination": 2.23×10^{-3}, "transcription from RNA polymerase II promoter": 3.64×10^{-3}, "embryonic hemopoiesis": 4.33×10^{-3}, "mitotic cell cycle spindle assembly checkpoint": 5.05×10^{-3}, "phosphoinositide-mediated signaling": 7.09×10^{-3}, "positive regulation of transcription, DNA-dependent": 7.39×10^{-3}

9	BIOPOLYMER_METABOLIC_PROCESS	C5	1	Genes annotated by the GO term GO:0043283. The chemical reactions and pathways involving biopolymers, long, repeating chains of monomers found in nature e.g. polysaccharides and proteins.	"regulation of transcription from RNA polymerase II promoter": $< 2.22\times10^{-16}$, "transcription from RNA polymerase II promoter": $< 2.22\times10^{-16}$, "protein amino acid phosphorylation": $< 2.22\times10^{-16}$, "negative regulation of transcription from RNA polymerase II promoter": $< 2.22\times10^{-16}$, "RNA splicing": $< 2.22\times10^{-16}$, "positive regulation of transcription from RNA polymerase II promoter": $< 2.22\times10^{-16}$, "DNA repair": $< 2.22\times10^{-16}$, "protein amino acid dephosphorylation": $< 2.22\times10^{-16}$, "protein modification process": $< 2.22\times10^{-16}$, "ubiquitin-dependent protein catabolic process": $< 2.22\times10^{-16}$, "DNA replication": $< 2.22\times10^{-16}$, "transcription initiation from RNA polymerase II promoter": $< 2.22\times10^{-16}$, "mRNA processing": $< 2.22\times10^{-16}$, "regulation of transcription, DNA-dependent": $< 2.22\times10^{-16}$, "positive regulation of transcription, DNA-dependent": 2.37×10^{-15}, "protein amino acid autophosphorylation": 2.71×10^{-15}, "protein amino acid glycosylation": 1.89×10^{-13}, "negative regulation of transcription, DNA-dependent": 1.27×10^{-12}, "RNA processing": 1.94×10^{-11}, "RNA elongation from RNA polymerase II promoter": 4.55×10^{-11}, "nuclear mRNA splicing, via spliceosome": 4.41×10^{-9}, "reciprocal meiotic recombination": 7.14×10^{-9}, "DNA recombination": 1.06×10^{-7}, "DNA damage checkpoint": 1.25×10^{-7}, "protein ubiquitination": 3.11×10^{-7}, "protein amino acid O-linked glycosylation": 3.18×10^{-7}, "negative regulation of DNA replication": 1.13×10^{-6}, "mRNA splice site selection": 3.99×10^{-6}, "spliceosome assembly": 7.22×10^{-6}, "DNA-dependent DNA replication": 1.41×10^{-5}, "L-fucose catabolic process": 1.41×10^{-5}, "protein kinase cascade": 2.01×10^{-5}, "nucleotide-excision repair, DNA damage removal": 3.56×10^{-5}, "transcription from RNA polymerase III promoter": 1.27×10^{-4}, "protein amino acid ADP-ribosylation": 1.27×10^{-4}, "androgen receptor signaling pathway": 2.26×10^{-4}, "telomere maintenance via telomerase": 6.27×10^{-4}, "protein amino acid N-linked glycosylation": 1.30×10^{-3}, "spliceosomal snRNP biogenesis": 1.61×10^{-3}, "nucleosome disassembly": 2.22×10^{-3}, "mRNA polyadenylation": 2.22×10^{-3}, "protein processing": 2.22×10^{-3}, "response to DNA damage stimulus": 2.78×10^{-3}, "telomere maintenance": 6.93×10^{-3}, "DNA fragmentation during apoptosis": 6.93×10^{-3}, "DNA replication initiation": 6.93×10^{-3}, "ER-associated protein catabolic process": 7.86×10^{-3}, "nucleotide-excision repair": 7.86×10^{-3}, "B cell lineage commitment": 7.86×10^{-3}

10	INTRACELLULAR_ORGANELLE_PART	C5	-1	Genes annotated by the GO term GO:0044446. A constituent part of an intracellular organelle, an organized structure of distinctive morphology and function, occurring within the cell. Includes constituent parts of the nucleus, mitochondria, plastids, vacuoles, vesicles, ribosomes and the cytoskeleton but excludes the plasma membrane.	"RNA splicing": $< 2.22\times10^{-16}$, "transcription initiation from RNA polymerase II promoter": $< 2.22\times10^{-16}$, "transcription, DNA-dependent": 2.13×10^{-8}, "nuclear mRNA splicing, via spliceosome": 3.60×10^{-8}, "mitosis": 1.32×10^{-7}, "protein targeting to mitochondrion": 3.03×10^{-7}, "telomere maintenance via telomerase": 7.07×10^{-7}, "RNA elongation from RNA polymerase II promoter": 2.83×10^{-6}, "protein amino acid N-linked glycosylation via asparagine": 4.06×10^{-6}, "microtubule cytoskeleton organization": 4.06×10^{-6}, "positive regulation of transcription from RNA polymerase II promoter": 1.21×10^{-5}, "nucleotide-excision repair, DNA gap filling": 2.09×10^{-5}, "centrosome organization": 2.34×10^{-5}, "retrograde vesicle-mediated transport, Golgi to ER": 5.88×10^{-5}, "spliceosomal snRNP biogenesis": 9.61×10^{-5}, "snRNA processing": 1.34×10^{-4}, "negative regulation of microtubule depolymerization": 1.34×10^{-4}, "protein complex assembly": 2.50×10^{-4}, "mitotic cell cycle spindle assembly checkpoint": 2.83×10^{-4}, "telomere maintenance": 3.00×10^{-4}, "response to DNA damage stimulus": 5.58×10^{-4}, "mitotic spindle organization": 1.07×10^{-3}, "androgen receptor signaling pathway": 1.19×10^{-3}, "microtubule nucleation": 1.36×10^{-3}, "intracellular transport": 3.78×10^{-3}, "sequestering of calcium ion": 4.45×10^{-3}, "histone deacetylation": 4.45×10^{-3}, "antigen processing and presentation of endogenous peptide antigen via MHC class I": 4.45×10^{-3}, "mitochondrion transport along microtubule": 4.45×10^{-3}, "preassembly of GPI anchor in ER membrane": 6.05×10^{-3}, "double-strand break repair via nonhomologous end joining": 7.16×10^{-3}, "cellular homeostasis": 7.16×10^{-3}, "mitotic sister chromatid segregation": 7.16×10^{-3}, "positive regulation of stress fiber formation": 7.41×10^{-3}, "chromosome segregation": 9.49×10^{-3}, "actin filament-based movement": 9.49×10^{-3}

Table 5: set.pcs

#	Set	Cat.	Sign	MSigDB Description	Enriched GO BP Terms (adj. p-value)
1	GNF2_CCNA2	C4	−1	Neighborhood of CCNA2	"phosphoinositide-mediated signaling": $< 2.22\times10^{-16}$, "mitotic chromosome condensation": 4.35×10^{-14}, "DNA replication": 1.01×10^{-12}, "spindle organization": 1.37×10^{-9}, "establishment of mitotic spindle localization": 9.59×10^{-5}, "kinetochore assembly": 4.76×10^{-4}, "DNA repair": 5.78×10^{-3}, "mitosis": 9.44×10^{-3}
2	GNF2_PCNA	C4	−1	Neighborhood of PCNA	"phosphoinositide-mediated signaling": $< 2.22\times10^{-16}$, "DNA replication": 1.47×10^{-15}, "mitotic chromosome condensation": 2.36×10^{-7}, "spindle organization": 4.33×10^{-7}, "establishment of mitotic spindle localization": 9.59×10^{-5}, "cell proliferation": 4.18×10^{-4}, "DNA repair": 4.33×10^{-4}, "kinetochore assembly": 4.76×10^{-4}, "mitosis": 9.44×10^{-3}
3	GNF2_CDC20	C4	−1	Neighborhood of CDC20	"phosphoinositide-mediated signaling": $< 2.22\times10^{-16}$, "spindle organization": 2.20×10^{-12}, "mitotic cell cycle spindle assembly checkpoint": 4.07×10^{-11}, "mitotic chromosome condensation": 1.52×10^{-9}, "cell proliferation": 8.96×10^{-9}, "mitosis": 1.83×10^{-8}, "establishment of mitotic spindle localization": 8.95×10^{-5}, "kinetochore assembly": 4.45×10^{-4}, "DNA replication": 7.83×10^{-3}
4	module_54	C4	−1	Genes in module_54	"DNA replication": $< 2.22\times10^{-16}$, "regulation of cyclin-dependent protein kinase activity": $< 2.22\times10^{-16}$, "phosphoinositide-mediated signaling": 9.52×10^{-14}, "nucleotide-excision repair, DNA gap filling": 4.86×10^{-7}, "mitotic chromosome condensation": 6.06×10^{-7}, "DNA repair": 2.17×10^{-6}, "mitotic cell cycle spindle assembly checkpoint": 2.81×10^{-6}, "cell proliferation": 6.29×10^{-6}, "G1 phase of mitotic cell cycle": 1.08×10^{-4}, "regulation of mitosis": 3.22×10^{-4}, "spindle organization": 6.66×10^{-4}, "mitotic sister chromatid segregation": 3.50×10^{-3}
5	GNF2_HMMR	C4	−1	Neighborhood of HMMR	"phosphoinositide-mediated signaling": $< 2.22\times10^{-16}$, "mitotic cell cycle spindle assembly checkpoint": 1.26×10^{-11}, "spindle organization": 4.89×10^{-10}, "mitotic chromosome condensation": 8.46×10^{-8}, "cell proliferation": 6.22×10^{-6}, "DNA replication": 1.09×10^{-5}, "establishment of mitotic spindle localization": 5.33×10^{-5}, "kinetochore assembly": 2.65×10^{-4}, "protein complex localization": 8.29×10^{-3}, "regulation of striated muscle development": 8.29×10^{-3}, "metaphase plate congression": 8.29×10^{-3}
6	GNF2_RRM1	C4	−1	Neighborhood of RRM1	"phosphoinositide-mediated signaling": $< 2.22\times10^{-16}$, "DNA replication": 5.95×10^{-14}, "spindle organization": 5.47×10^{-9}, "mitotic chromosome condensation": 9.33×10^{-7}, "fumarate metabolic process": 2.11×10^{-4}, "regulation of mRNA stability": 2.33×10^{-4}, "kinetochore assembly": 1.05×10^{-3}
7	GNF2_MKI67	C4	−1	Neighborhood of MKI67	"phosphoinositide-mediated signaling": 1.95×10^{-10}, "spindle organization": 5.86×10^{-6}, "establishment of mitotic spindle localization": 1.10×10^{-5}, "kinetochore assembly": 5.48×10^{-5}, "mitotic chromosome condensation": 1.37×10^{-4}, "protein complex localization": 2.55×10^{-3}, "regulation of striated muscle development": 2.55×10^{-3}, "metaphase plate congression": 2.55×10^{-3}
8	GNF2_TTK	C4	−1	Neighborhood of TTK	"phosphoinositide-mediated signaling": 4.05×10^{-16}, "DNA replication": 1.04×10^{-9}, "mitotic chromosome condensation": 1.32×10^{-8}, "regulation of striated muscle development": 3.76×10^{-3}, "metaphase plate congression": 3.76×10^{-3}

9	GNF2_CDC2	C4	−1	Neighborhood of CDC2	"phosphoinositide-mediated signaling": $< 2.22\times10^{-16}$, "mitotic chromosome condensation": 3.28×10^{-12}, "spindle organization": 2.08×10^{-7}, "DNA repair": 8.04×10^{-6}, "nucleosome assembly": 2.21×10^{-5}, "kinetochore assembly": 2.92×10^{-4}, "DNA replication": 3.23×10^{-3}, "protein complex localization": 8.92×10^{-3}, "DNA strand elongation during DNA replication": 8.92×10^{-3}, "regulation of striated muscle development": 8.92×10^{-3}, "metaphase plate congression": 8.92×10^{-3}
10	GNF2_CENPF	C4	−1	Neighborhood of CENPF	"phosphoinositide-mediated signaling": $< 2.22\times10^{-16}$, "DNA replication": 2.86×10^{-8}, "mitotic chromosome condensation": 8.17×10^{-8}, "spindle organization": 1.74×10^{-7}, "kinetochore assembly": 2.60×10^{-4}, "cell cycle": 6.73×10^{-3}, "protein complex localization": 8.17×10^{-3}, "regulation of striated muscle development": 8.17×10^{-3}, "metaphase plate congression": 8.17×10^{-3}

Table 6: set.t.stat

#	Set	Cat.	Sign	MSigDB Description	Enriched GO BP Terms (adj. p-value)
1	GNF2_PCNA	C4	−1	Neighborhood of PCNA	"phosphoinositide-mediated signaling": $< 2.22\times10^{-16}$, "DNA replication": 1.47×10^{-15}, "mitotic chromosome condensation": 2.36×10^{-7}, "spindle organization": 4.33×10^{-7}, "establishment of mitotic spindle localization": 9.59×10^{-5}, "cell proliferation": 4.18×10^{-4}, "DNA repair": 4.33×10^{-4}, "kinetochore assembly": 4.76×10^{-4}, "mitosis": 9.44×10^{-3}
2	GNF2_CCNA2	C4	−1	Neighborhood of CCNA2	"phosphoinositide-mediated signaling": $< 2.22\times10^{-16}$, "mitotic chromosome condensation": 4.35×10^{-14}, "DNA replication": 1.01×10^{-12}, "spindle organization": 1.37×10^{-9}, "establishment of mitotic spindle localization": 9.59×10^{-5}, "kinetochore assembly": 4.76×10^{-4}, "DNA repair": 5.78×10^{-3}, "mitosis": 9.44×10^{-3}
3	GNF2_CENPF	C4	−1	Neighborhood of CENPF	"phosphoinositide-mediated signaling": $< 2.22\times10^{-16}$, "DNA replication": 2.86×10^{-8}, "mitotic chromosome condensation": 8.17×10^{-8}, "spindle organization": 1.74×10^{-7}, "kinetochore assembly": 2.60×10^{-4}, "cell cycle": 6.73×10^{-3}, "protein complex localization": 8.17×10^{-3}, "regulation of striated muscle development": 8.17×10^{-3}, "metaphase plate congression": 8.17×10^{-3}
4	GNF2_CCNB2	C4	−1	Neighborhood of CCNB2	"phosphoinositide-mediated signaling": $< 2.22\times10^{-16}$, "spindle organization": 2.44×10^{-12}, "mitotic cell cycle spindle assembly checkpoint": 4.58×10^{-11}, "mitotic chromosome condensation": 1.68×10^{-9}, "DNA replication": 1.83×10^{-7}, "mitosis": 1.80×10^{-5}, "establishment of mitotic spindle localization": 9.42×10^{-5}, "kinetochore assembly": 4.68×10^{-4}, "cell proliferation": 2.63×10^{-3}
5	GNF2_CDC2	C4	−1	Neighborhood of CDC2	"phosphoinositide-mediated signaling": $< 2.22\times10^{-16}$, "mitotic chromosome condensation": 3.28×10^{-12}, "spindle organization": 2.08×10^{-7}, "DNA repair": 8.04×10^{-6}, "nucleosome assembly": 2.21×10^{-5}, "kinetochore assembly": 2.92×10^{-4}, "DNA replication": 3.23×10^{-3}, "protein complex localization": 8.92×10^{-3}, "DNA strand elongation during DNA replication": 8.92×10^{-3}, "regulation of striated muscle development": 8.92×10^{-3}, "metaphase plate congression": 8.92×10^{-3}
6	GNF2_CDC20	C4	1	Neighborhood of CDC20	"phosphoinositide-mediated signaling": $< 2.22\times10^{-16}$, "spindle organization": 2.20×10^{-12}, "mitotic cell cycle spindle assembly checkpoint": 4.07×10^{-11}, "mitotic chromosome condensation": 1.52×10^{-9}, "cell proliferation": 8.96×10^{-9}, "mitosis": 1.83×10^{-8}, "establishment of mitotic spindle localization": 8.95×10^{-5}, "kinetochore assembly": 4.45×10^{-4}, "DNA replication": 7.83×10^{-3}
7	GNF2_RRM1	C4	−1	Neighborhood of RRM1	"phosphoinositide-mediated signaling": $< 2.22\times10^{-16}$, "DNA replication": 5.95×10^{-14}, "spindle organization": 5.47×10^{-9}, "mitotic chromosome condensation": 9.33×10^{-7}, "fumarate metabolic process": 2.11×10^{-4}, "regulation of mRNA stability": 2.33×10^{-4}, "kinetochore assembly": 1.05×10^{-3}
8	module_54	C4	−1	Genes in module_54	"DNA replication": $< 2.22\times10^{-16}$, "regulation of cyclin-dependent protein kinase activity": $< 2.22\times10^{-16}$, "phosphoinositide-mediated signaling": 9.52×10^{-14}, "nucleotide-excision repair, DNA gap filling": 4.86×10^{-7}, "mitotic chromosome condensation": 6.06×10^{-7}, "DNA repair": 2.17×10^{-6}, "mitotic cell cycle spindle assembly checkpoint": 2.81×10^{-6}, "cell proliferation": 6.29×10^{-6}, "G1 phase of mitotic cell cycle": 1.08×10^{-4}, "regulation of mitosis": 3.22×10^{-4}, "spindle organization": 6.66×10^{-4}, "mitotic sister chromatid segregation": 3.50×10^{-3}

9	GNF2_RFC4	C4	-1	Neighborhood of RFC4	"DNA replication": $< 2.22\times10^{-16}$, "phosphoinositide-mediated signaling": 9.91×10^{-14}, "nucleotide-excision repair, DNA gap filling": 1.09×10^{-10}, "DNA repair": 2.87×10^{-6}, "mitotic chromosome condensation": 3.19×10^{-5}, "maintenance of DNA repeat elements": 1.68×10^{-3}, "positive regulation of helicase activity": 7.74×10^{-3}
10	module_292	C4	-1	Genes in module_292	"immune response": $< 2.22\times10^{-16}$, "T cell costimulation": 5.42×10^{-8}, "regulation of cytokine-mediated signaling pathway": 2.60×10^{-6}, "immune response-regulating cell surface receptor signaling pathway": 2.60×10^{-6}, "B cell receptor transport into membrane raft": 2.60×10^{-6}, "negative regulation of transforming growth factor-beta3 production": 2.60×10^{-6}, "regulation of epithelial cell differentiation": 1.78×10^{-5}, "response to molecule of bacterial origin": 7.01×10^{-5}, "chronic inflammatory response": 1.24×10^{-4}, "negative regulation of antigen processing and presentation of peptide or polysaccharide antigen via MHC class II": 1.24×10^{-4}, "negative regulation of dendritic cell antigen processing and presentation": 1.24×10^{-4}, "positive regulation of oxygen and reactive oxygen species metabolic process": 1.24×10^{-4}, "negative regulation of plasma membrane long-chain fatty acid transport": 1.24×10^{-4}, "negative regulation of nitric oxide mediated signal transduction": 1.24×10^{-4}, "negative regulation of cGMP-mediated signaling": 1.24×10^{-4}, "negative regulation of plasminogen activation": 1.24×10^{-4}, "positive regulation of macrophage chemotaxis": 1.24×10^{-4}, "response to magnesium ion": 1.24×10^{-4}, "response to progesterone stimulus": 1.24×10^{-4}, "negative regulation of interleukin-12 production": 1.24×10^{-4}, "negative regulation of fibroblast growth factor receptor signaling pathway": 1.24×10^{-4}, "engulfment of apoptotic cell": 1.24×10^{-4}, "regulation of MAPKKK cascade": 5.07×10^{-4}, "Wnt receptor signaling pathway": 5.07×10^{-4}, "sprouting angiogenesis": 7.32×10^{-4}, "cellular response to heat": 7.32×10^{-4}, "cell activation": 2.15×10^{-3}, "positive regulation of fibroblast cell migration": 2.52×10^{-3}, "positive regulation of activated T cell proliferation": 3.93×10^{-3}, "response to glucose stimulus": 6.59×10^{-3}, "positive regulation of transforming growth factor-beta1 production": 6.59×10^{-3}, "negative regulation of fibrinolysis": 6.59×10^{-3}

Table 7: set.u.stat.pval.log

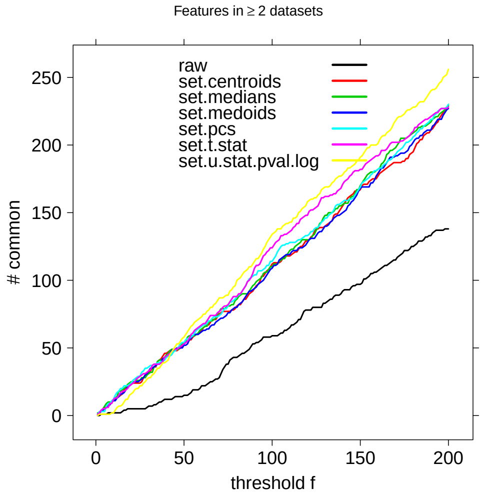

(a) ≥ 2 datasets

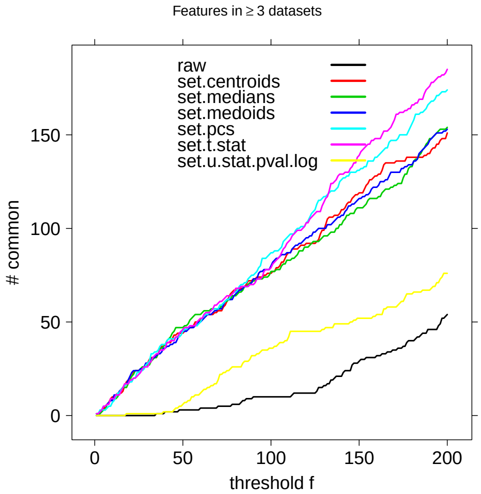

(b) ≥ 3 datasets

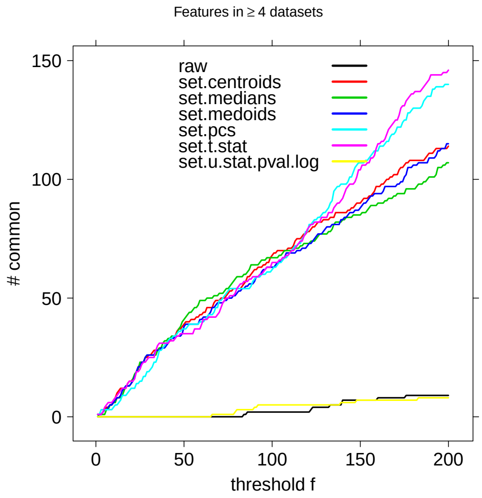

(c) ≥ 4 datasets

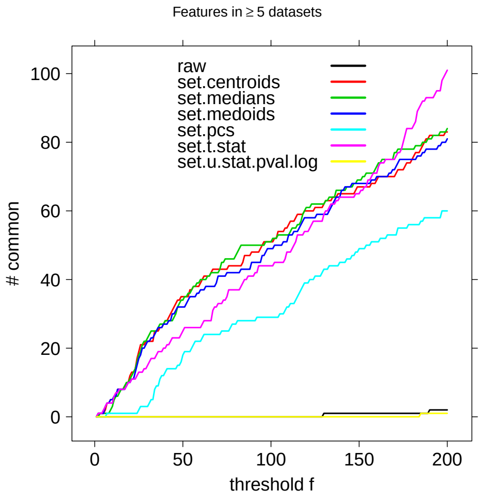

(d) ≥ 5 datasets

Figure 7: Concordance between lists for different levels of cutoffs f

3.4 Concordance

Figure 7 shows concordance between lists produced by the different datasets, for $k = 2, 3, 4, 5$.

3.5 Bootstrap

Figure 8 shows the variability of ranks evaluated using the bootstrap procedure, for different set statistics.

3.6 Kolmogorov-Smirnov Enrichmnent for MSigDB Categories

Figure 9 shows the Kolmogorov-Smirnov Brownian-bridge plots for different set statistics.

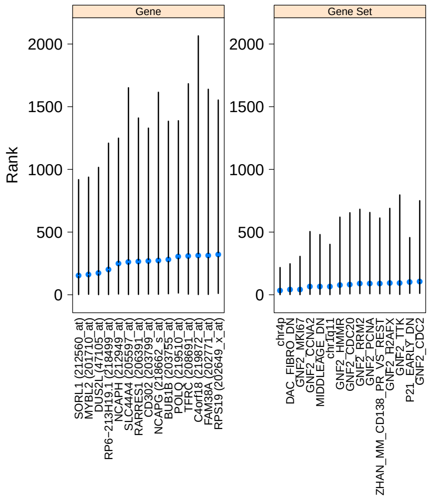

(a) set centroid

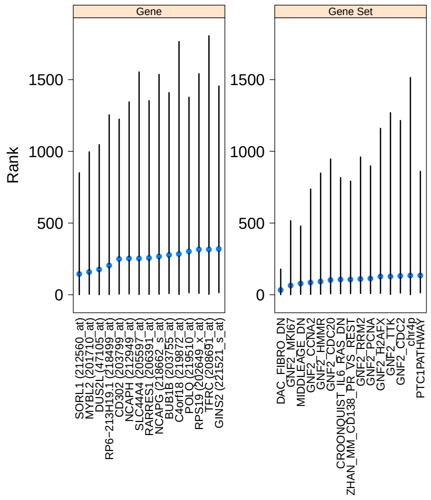

(b) set medoid

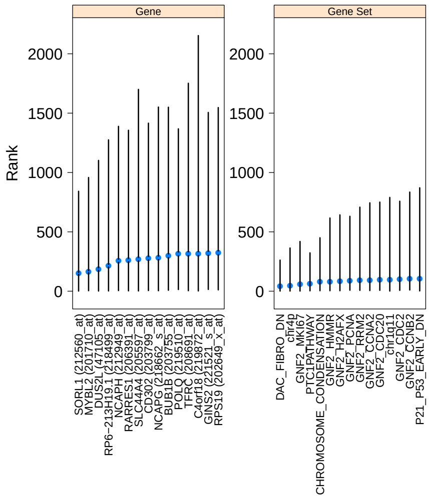

(c) set median

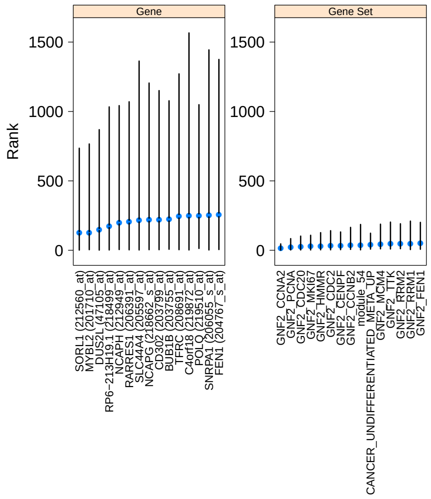

(d) set t statistic

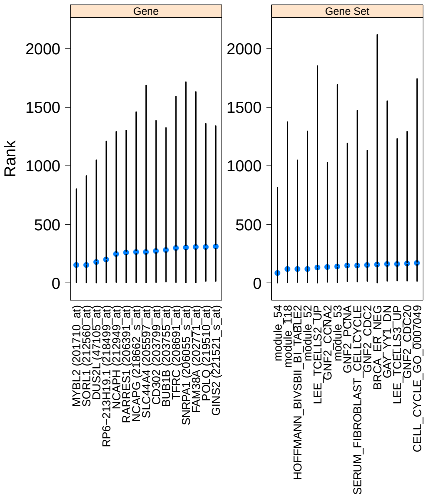

(e) set PC

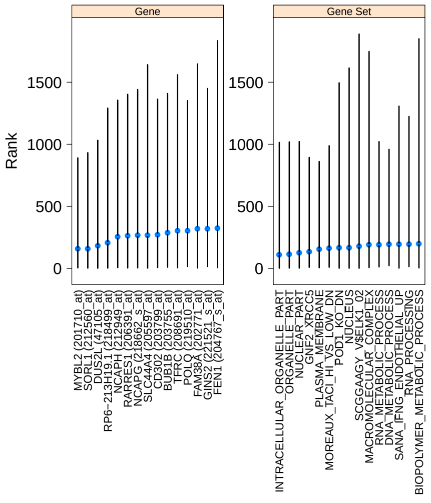

(f) set U statistic

Figure 8

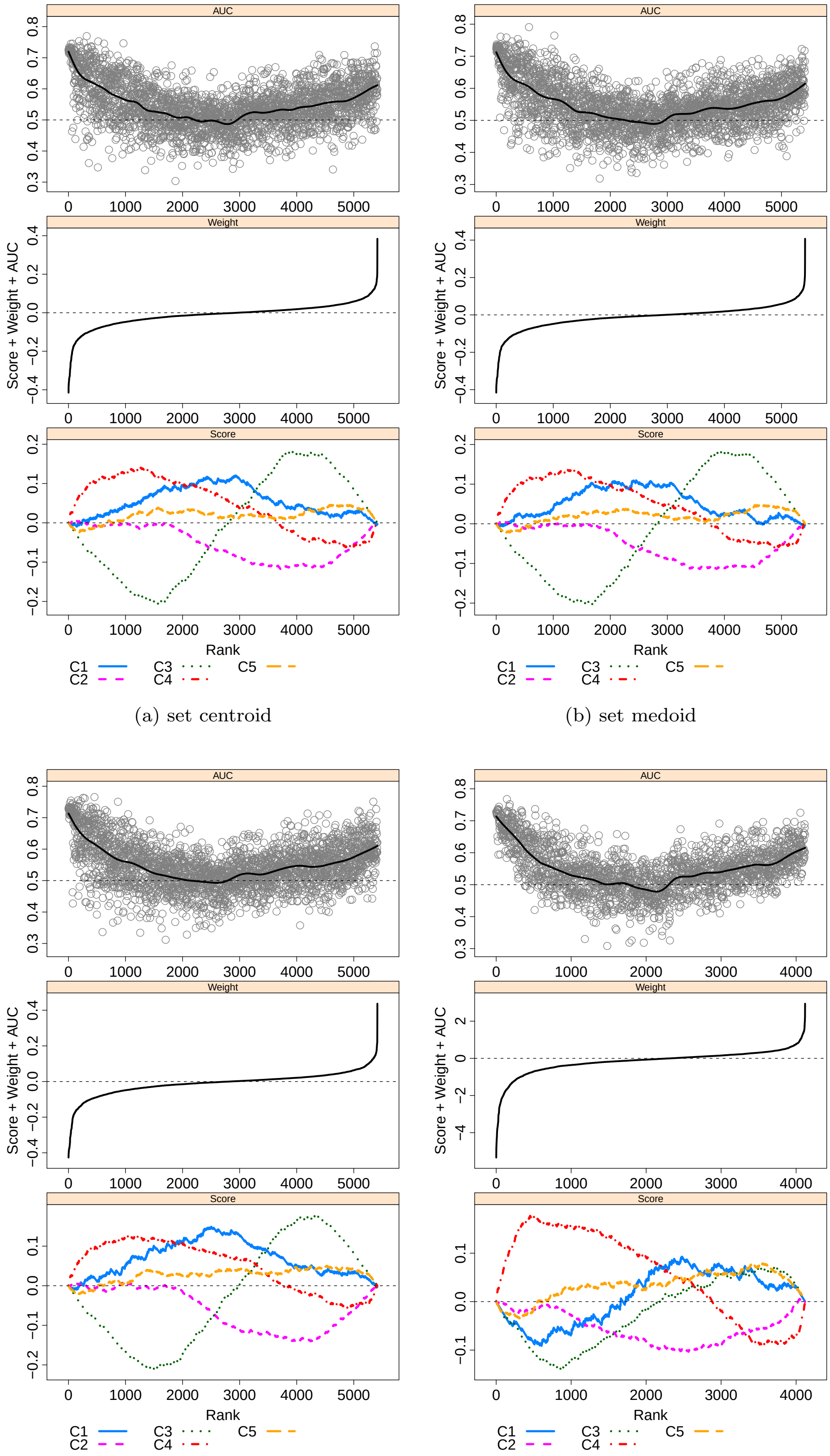

(a) set centroid

(b) set medoid

(c) set median

(d) set t statistic

Figure 9: Kolmogorov-Smirnov Brownian-bridge plots

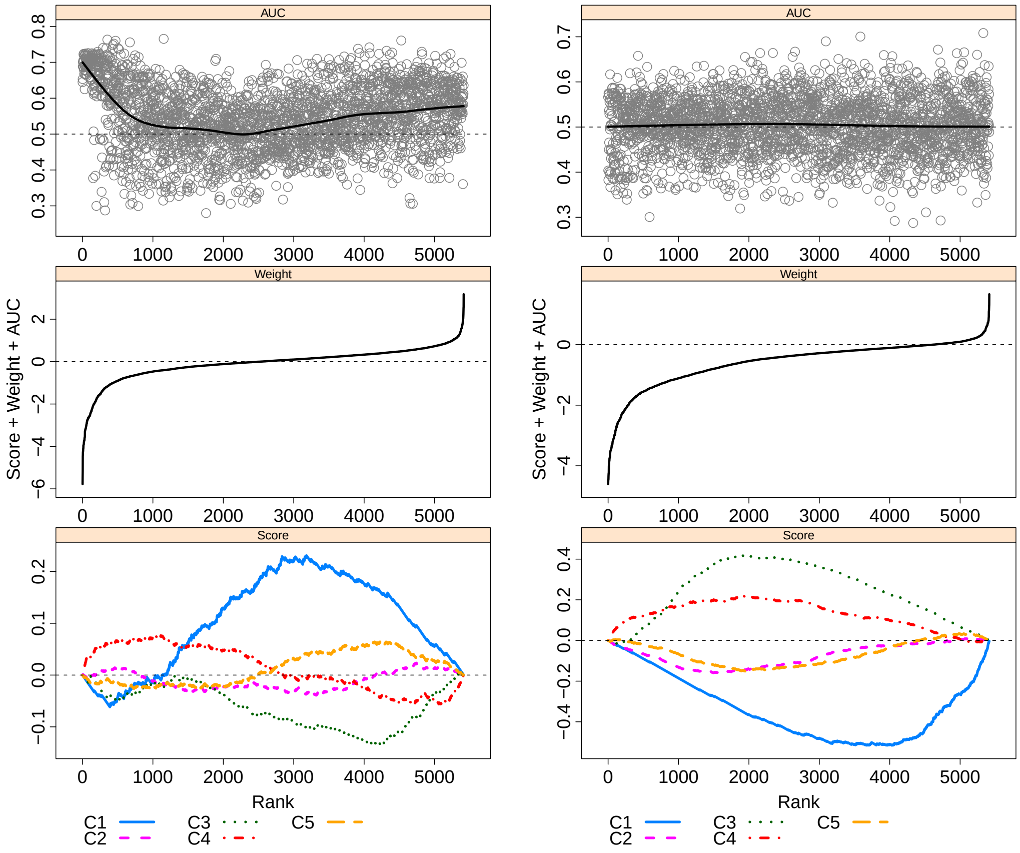

(e) set PC

(f) set PC

Figure 9: Kolmogorov-Smirnov Brownian-bridge plots

3.7 Effect of Set Size on AUC and Centroid Weights

Figure 10 shows the AUC and centroid classifier weights as functions of set size, for the set centroid statistic. There does not appear to be a simple monotonic relationship between the size of the gene set and its centroid classifier weight or its AUC.

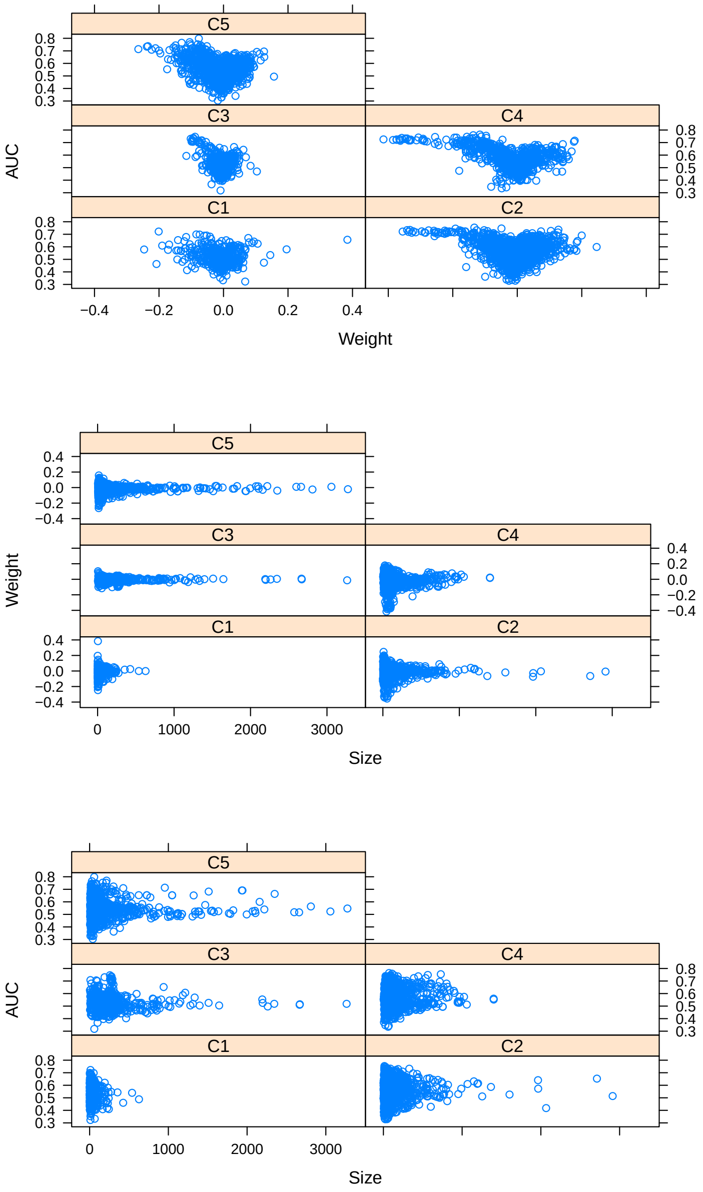

Figure 10: AUC and weight versus set size for set centroid statistic.

3.8 Top Gene Sets for each ER/HER2 Subtype

Tables 8, 9, 10, 11, 12, 13 show the top 10 MSigDB sets for ER/HER2 subtypes, chosen by the centroid classifier. Sign=−1 if expression is negatively associated with long-term survival, and vice versa.

Figure 11 shows Kolmogorov-Smirnov plots for each set statistic in each ER/HER2 subtype. The sets were sorted in increasing order of centroid classifier weight. For each subtype, the line in the plots moves up one step of size $1/m$ (m is number of sets containing at least one of Desmedt's genes) when the set contains at least one genes belonging to a module defined in [24] and down when not (step size of $1/n$ where n is number of sets not containing at least one genes). Each molecular subgroup uses a different set of Desmedt's module genes: HER2+ is compared against modules PLAU and STAT1, ER−/HER2− against module STAT1, and ER+/HER2− against module AURKA.

Class	#	MSigDB Set	Cat.	Description	Sign
ER−/HER2−	1	chr7q12	C1	Genes in cytogenetic band chr7q12	1
	2	COLLER_MYC_DN	C2	Genes down-regulated by MYC in 293T (transformed fetal renal cell).	−1
	3	IFNGPATHWAY	C2	IFN gamma signaling pathway	1
	4	GRANDVAUX_IFN_NOT_IRF3_UP	C2	Genes up-regulated by interferon-alpha,beta but not by IRF3 in Jurkat (T cell)	1
	5	GNF2_ST13	C4	Neighborhood of ST13	−1
	6	GNF2_CD48	C4	Neighborhood of CD48	1
	7	GNF2_GLTSCR2	C4	Neighborhood of GLTSCR2	−1
	8	MENSE_HYPOXIA_DN	C2	List of Hypoxia-suppressed genes found in both Astrocytes and HeLa Cells	−1
	9	HSA03010_RIBOSOME	C2	Genes involved in ribosome	−1
	10	GCM_TPT1	C4	Neighborhood of TPT1	−1
ER+/HER2−	1	GNF2_MKI67	C4	Neighborhood of MKI67	−1
	2	GNF2_TTK	C4	Neighborhood of TTK	−1
	3	GNF2_HMMR	C4	Neighborhood of HMMR	−1
	4	GNF2_CCNA2	C4	Neighborhood of CCNA2	−1
	5	GNF2_SMC2L1	C4	Neighborhood of SMC2L1	−1
	6	GNF2_ESPL1	C4	Neighborhood of ESPL1	−1
	7	GNF2_CDC20	C4	Neighborhood of CDC20	−1
	8	GNF2_H2AFX	C4	Neighborhood of H2AFX	−1
	9	GNF2_RRM2	C4	Neighborhood of RRM2	−1
	10	ZHAN_MM_CD138_PR_VS_REST	C2	50 top ranked SAM-defined over-expressed genes in each subgroup__PR	−1
HER2+	1	chr4p	C1	Genes in cytogenetic band chr4p	−1
	2	chr1q11	C1	Genes in cytogenetic band chr1q11	1
	3	DAC_FIBRO_DN	C2	Downregulated by DAC treatment in LD419 fibroblast cells	−1
	4	GNF2_MKI67	C4	Neighborhood of MKI67	−1
	5	GNF2_CCNA2	C4	Neighborhood of CCNA2	−1
	6	GNF2_TTK	C4	Neighborhood of TTK	−1
	7	GNF2_H2AFX	C4	Neighborhood of H2AFX	−1
	8	GNF2_HMMR	C4	Neighborhood of HMMR	−1
	9	CROONQUIST_IL6_RAS_DN	C2	Genes dowmregulated in multiple myeloma cells exposed to the pro-proliferative cytokine IL-6 versus those with N-ras-activating mutations.	−1

10	CROONQUIST_IL6_STARVE_UP	C2	Genes upregulated in multiple myeloma cells exposed to the pro-proliferative cytokine IL-6 versus those that were IL-6-starved.	−1

Table 8: set.centroids

Class	#	MSigDB Set	Cat.	Description	Sign
ER−/HER2−	1	GRANDVAUX_IFN_NOT_IRF3_UP	C2	Genes up-regulated by interferon-alpha,beta but not by IRF3 in Jurkat (T cell)	1
	2	chr7q12	C1	Genes in cytogenetic band chr7q12	1
	3	module_144	C4	Genes in module_144	1
	4	module_293	C4	Genes in module_293	1
	5	1_AND_2_METHYLNAPHTHALENE_DEGRADATION	C2		−1
	6	chr3q23	C1	Genes in cytogenetic band chr3q23	−1
	7	GNF2_CD48	C4	Neighborhood of CD48	1
	8	HSA00511_N_GLYCAN_DEGRADATION	C2	Genes involved in N-glycan degradation	1
	9	GNF2_ST13	C4	Neighborhood of ST13	−1
	10	BLYMPHOCYTEPATHWAY	C2	B cells express the major histocompatibility complex (class II MHC), immunoglobulins, adhesion proteins, and other factors on their cell surface.	1
ER+/HER2−	1	GNF2_MKI67	C4	Neighborhood of MKI67	−1
	2	GNF2_HMMR	C4	Neighborhood of HMMR	−1
	3	P21_P53_EARLY_DN	C2	Down-regulated at early timepoints (4-8 hrs) following ectopic expression of p21 (CDKN1A) in OvCa cells, p53-dependent	−1
	4	GNF2_TTK	C4	Neighborhood of TTK	−1
	5	GNF2_SMC2L1	C4	Neighborhood of SMC2L1	−1
	6	ZHAN_MM_CD138_PR_VS_REST	C2	50 top ranked SAM-defined overexpressed genes in each subgroup__PR	−1
	7	chr1q11	C1	Genes in cytogenetic band chr1q11	1
	8	GNF2_CDC20	C4	Neighborhood of CDC20	−1
	9	GNF2_ESPL1	C4	Neighborhood of ESPL1	−1
	10	GNF2_RRM2	C4	Neighborhood of RRM2	−1
HER2+	1	chr4p	C1	Genes in cytogenetic band chr4p	−1
	2	chr1q11	C1	Genes in cytogenetic band chr1q11	1
	3	DAC_FIBRO_DN	C2	Downregulated by DAC treatment in LD419 fibroblast cells	−1
	4	GNF2_MKI67	C4	Neighborhood of MKI67	−1
	5	GNF2_HMMR	C4	Neighborhood of HMMR	−1
	6	GNF2_H2AFX	C4	Neighborhood of H2AFX	−1
	7	GNF2_TTK	C4	Neighborhood of TTK	−1
	8	GNF2_CCNA2	C4	Neighborhood of CCNA2	−1
	9	CROONQUIST_IL6_STARVE_UP	C2	Genes upregulated in multiple myeloma cells exposed to the pro-proliferative cytokine IL-6 versus those that were IL-6-starved.	−1
	10	GNF2_PCNA	C4	Neighborhood of PCNA	−1

Table 9: set.medians

Class	#	MSigDB Set	Cat.	Description	Sign
ER−/HER2−	1	chr7q12	C1	Genes in cytogenetic band chr7q12	1
	2	IFNGPATHWAY	C2	IFN gamma signaling pathway	1
	3	chr19p11	C1	Genes in cytogenetic band chr19p11	1
	4	1_AND_2_METHYLNAPHTHALENE_DEGRADATION	C2		−1
	5	GRANDVAUX_IFN_NOT_IRF3_UP	C2	Genes up-regulated by interferon-alpha,beta but not by IRF3 in Jurkat (T cell)	1
	6	GNF2_ST13	C4	Neighborhood of ST13	−1
	7	COLLER_MYC_DN	C2	Genes down-regulated by MYC in 293T (transformed fetal renal cell).	−1
	8	HEDVAT_ELF_DN	C2	MEF Regulates IL-8 Expression, genes down-regulated by more than 2 fold	1
	9	GNF2_GLTSCR2	C4	Neighborhood of GLTSCR2	−1
	10	HSA03010_RIBOSOME	C2	Genes involved in ribosome	−1
ER+/HER2−	1	GNF2_MKI67	C4	Neighborhood of MKI67	−1
	2	GNF2_TTK	C4	Neighborhood of TTK	−1
	3	GNF2_HMMR	C4	Neighborhood of HMMR	−1
	4	chr1q11	C1	Genes in cytogenetic band chr1q11	1
	5	GNF2_SMC2L1	C4	Neighborhood of SMC2L1	−1
	6	GNF2_CCNA2	C4	Neighborhood of CCNA2	−1
	7	GNF2_ESPL1	C4	Neighborhood of ESPL1	−1
	8	GNF2_CDC20	C4	Neighborhood of CDC20	−1
	9	GNF2_H2AFX	C4	Neighborhood of H2AFX	−1
	10	ZHAN_MM_CD138_PR_VS_REST	C2	50 top ranked SAM-defined over-expressed genes in each subgroup__PR	−1
HER2+	1	chr4p	C1	Genes in cytogenetic band chr4p	−1
	2	chr1q11	C1	Genes in cytogenetic band chr1q11	1
	3	DAC_FIBRO_DN	C2	Downregulated by DAC treatment in LD419 fibroblast cells	−1
	4	GNF2_MKI67	C4	Neighborhood of MKI67	−1
	5	GNF2_CCNA2	C4	Neighborhood of CCNA2	−1
	6	CROONQUIST_IL6_RAS_DN	C2	Genes dowmregulated in multiple myeloma cells exposed to the pro-proliferative cytokine IL-6 versus those with N-ras-activating mutations.	−1
	7	GNF2_TTK	C4	Neighborhood of TTK	−1
	8	GNF2_H2AFX	C4	Neighborhood of H2AFX	−1
	9	chr13q	C1	Genes in cytogenetic band chr13q	1
	10	GNF2_HMMR	C4	Neighborhood of HMMR	−1

Table 10: set.medoids

Class	#	MSigDB Set	Cat.	Description	Sign
ER−/HER2−	1	TTGTTT_V$FOXO4_01	C3	Genes with promoter regions [-2kb,2kb] around transcription start site containing the motif TTGTTT which matches annotation for MLLT7: myeloid/lymphoid or mixed-lineage leukemia (trithorax homolog, Drosophila); translocated to, 7	−1
	2	AACTTT_UNKNOWN	C3	Genes with promoter regions [-2kb,2kb] around transcription start site containing motif AACTTT. Motif does not match any known transcription factor	−1
	3	TGGAAA_V$NFAT_Q4_01	C3	Genes with promoter regions [-2kb,2kb] around transcription start site containing the motif TGGAAA which matches annotation for NFAT¡br¿ NFATC	−1
	4	ALZHEIMERS_DISEASE_UP	C2	Upregulated in correlation with overt Alzheimer's Disease, in the CA1 region of the hippocampus	−1
	5	TATAAA_V$TATA_01	C3	Genes with promoter regions [-2kb,2kb] around transcription start site containing the motif TATAAA which matches annotation for TAF¡br¿ TATA	−1
	6	module_83	C4	Genes in module_83	−1
	7	MULTICELLULAR_ORGANISMAL_DEVELOPMENT	C5	Genes annotated by the GO term GO:0007275. The biological process whose specific outcome is the progression of an organism over time from an initial condition (e.g. a zygote or a young adult) to a later condition (e.g. a multicellular animal or an aged adult).	−1
	8	GNF2_EIF3S6	C4	Neighborhood of EIF3S6	−1
	9	ANATOMICAL_STRUCTURE_DEVELOPMENT	C5	Genes annotated by the GO term GO:0048856. The biological process whose specific outcome is the progression of an anatomical structure from an initial condition to its mature state. This process begins with the formation of the structure and ends with the mature structure, whatever form that may be including its natural destruction. An anatomical structure is any biological entity that occupies space and is distinguished from its surroundings. Anatomical structures can be macroscopic such as a carpel, or microscopic such as an acrosome.	−1
	10	GNF2_FBL	C4	Neighborhood of FBL	−1
ER+/HER2−	1	STEMCELL_NEURAL_UP	C2	Enriched in mouse neural stem cells, compared to differentiated brain and bone marrow cells	−1
	2	module_54	C4	Genes in module_54	−1

	3	STEMCELL_EMBRYONIC_UP	C2	Enriched in mouse embryonic stem cells, compared to differentiated brain and bone marrow cells	−1
	4	module_3	C4	Genes in module_3	−1
	5	TARTE_PLASMA_BLASTIC	C2	Genes overexpressed in mature plasma cells isolated from tonsils (TPCs) and mature plasma cells isolated from bone marrow (BMPCs) as compared to polyclonal plasmablastic cells (PPCs).	−1
	6	module_98	C4	Genes in module_98	−1
	7	module_52	C4	Genes in module_52	−1
	8	HOFFMANN_BIVSBII_BI_TABLE2	C2	Genes with at least five fold change in expression between Pre-BI and Large Pre-BII cells	−1
	9	GNF2_CCNA2	C4	Neighborhood of CCNA2	−1
	10	module_198	C4	Genes in module_198	−1
HER2+	1	module_54	C4	Genes in module_54	−1
	2	STEMCELL_NEURAL_UP	C2	Enriched in mouse neural stem cells, compared to differentiated brain and bone marrow cells	−1
	3	BOQUEST_CD31PLUS_VS_CD31MINUS_UP	C2	Genes overexpressed 3-fold or more in freshly isolated CD31+ versus freshly isolated CD31- cells	1
	4	module_3	C4	Genes in module_3	−1
	5	module_98	C4	Genes in module_98	−1
	6	TARTE_PLASMA_BLASTIC	C2	Genes overexpressed in mature plasma cells isolated from tonsils (TPCs) and mature plasma cells isolated from bone marrow (BMPCs) as compared to polyclonal plasmablastic cells (PPCs).	−1
	7	HOFFMANN_BIVSBII_BI_TABLE2	C2	Genes with at least five fold change in expression between Pre-BI and Large Pre-BII cells	−1
	8	STEMCELL_EMBRYONIC_UP	C2	Enriched in mouse embryonic stem cells, compared to differentiated brain and bone marrow cells	−1
	9	module_52	C4	Genes in module_52	−1
	10	module_198	C4	Genes in module_198	−1

Table 11: set.pcs

Class	#	MSigDB Set	Cat.	Description	Sign
ER−/HER2−	1	WIELAND_HEPATITIS_B_INDUCED	C2	Genes induced in the liver during hepatitis B viral clearance in chimpanzees.	1
	2	GNF2_EIF3S6	C4	Neighborhood of EIF3S6	−1
	3	GCM_TPT1	C4	Neighborhood of TPT1	−1
	4	HSA03010_RIBOSOME	C2	Genes involved in ribosome	−1
	5	STRUCTURAL_CONSTITUENT_OF_RIBOSOME	C5	Genes annotated by the GO term GO:0003735. The action of a molecule that contributes to the structural integrity of the ribosome.	−1
	6	RIBOSOMAL_PROTEINS	C2		−1
	7	GNF2_ST13	C4	Neighborhood of ST13	−1
	8	MORF_TPT1	C4	Neighborhood of TPT1	−1
	9	GNF2_TPT1	C4	Neighborhood of TPT1	−1
	10	GNF2_GLTSCR2	C4	Neighborhood of GLTSCR2	−1
ER+/HER2−	1	GNF2_CCNA2	C4	Neighborhood of CCNA2	−1
	2	module_54	C4	Genes in module_54	−1
	3	GNF2_PCNA	C4	Neighborhood of PCNA	−1
	4	GNF2_CDC20	C4	Neighborhood of CDC20	−1
	5	GNF2_HMMR	C4	Neighborhood of HMMR	−1
	6	GNF2_MKI67	C4	Neighborhood of MKI67	−1
	7	GNF2_RRM1	C4	Neighborhood of RRM1	−1
	8	GNF2_TTK	C4	Neighborhood of TTK	−1
	9	GNF2_CDC2	C4	Neighborhood of CDC2	−1
	10	GNF2_CCNB2	C4	Neighborhood of CCNB2	−1
HER2+	1	GNF2_CCNA2	C4	Neighborhood of CCNA2	−1
	2	GNF2_PCNA	C4	Neighborhood of PCNA	−1
	3	GNF2_CDC20	C4	Neighborhood of CDC20	−1
	4	GNF2_HMMR	C4	Neighborhood of HMMR	−1
	5	module_54	C4	Genes in module_54	−1
	6	GNF2_CENPF	C4	Neighborhood of CENPF	−1
	7	GNF2_CDC2	C4	Neighborhood of CDC2	−1
	8	GNF2_RRM1	C4	Neighborhood of RRM1	−1
	9	GNF2_MKI67	C4	Neighborhood of MKI67	−1
	10	GNF2_TTK	C4	Neighborhood of TTK	−1

Table 12: set.t.stat

Class	#	MSigDB Set	Cat.	Description	Sign
ER−/HER2−	1	BASSO_GERMINAL_CENTER_CD40_UP	C2	CD40 up-regulated genes	−1
	2	HSA04612_ANTIGEN_PROCESSING_AND_PRESENTATION	C2	Genes involved in antigen processing and presentation	−1
	3	module_45	C4	Genes in module_45	−1
	4	GNF2_CD53	C4	Neighborhood of CD53	−1
	5	module_119	C4	Genes in module_119	−1
	6	module_44	C4	Genes in module_44	−1
	7	FLECHNER_KIDNEY_TRANSPLANT_REJECTION_UP	C2	Genes upreglated in acute rejection transplanted kidney biopsies relative to well functioning transplanted kidney biopsies from stable, immunosuppressed, recipients (median FDR ¡ 0.14% per comparison)	−1
	8	module_292	C4	Genes in module_292	−1
	9	IMMUNE_RESPONSE	C5	Genes annotated by the GO term GO:0006955. Any immune system process that functions in the calibrated response of an organism to a potential internal or invasive threat.	−1
	10	IMMUNE_SYSTEM_PROCESS	C5	Genes annotated by the GO term GO:0002376. Any process involved in the development or functioning of the immune system, an organismal system for calibrated responses to potential internal or invasive threats.	−1
ER+/HER2−	1	GGGAGGRR_V$MAZ_Q6	C3	Genes with promoter regions [-2kb,2kb] around transcription start site containing the motif GGGAGGRR which matches annotation for MAZ: MYC-associated zinc finger protein (purine-binding transcription factor)	−1
	2	MORF_NME2	C4	Neighborhood of NME2	−1
	3	GGGTGGRR_V$PAX4_03	C3	Genes with promoter regions [-2kb,2kb] around transcription start site containing the motif GGGTGGRR which matches annotation for PAX4: paired box gene 4	−1
	4	STRUCTURAL_CONSTITUENT_OF_RIBOSOME	C5	Genes annotated by the GO term GO:0003735. The action of a molecule that contributes to the structural integrity of the ribosome.	−1
	5	TTGTTT_V$FOXO4_01	C3	Genes with promoter regions [-2kb,2kb] around transcription start site containing the motif TTGTTT which matches annotation for MLLT7: myeloid/lymphoid or mixed-lineage leukemia (trithorax homolog, Drosophila); translocated to, 7	−1

	6	TGGAAA_V$NFAT_Q4_01	C3	Genes with promoter regions [-2kb,2kb] around transcription start site containing the motif TGGAAA which matches annotation for NFAT¡br¿ NFATC	−1
	7	CAGGTG_V$E12_Q6	C3	Genes with promoter regions [-2kb,2kb] around transcription start site containing the motif CAGGTG which matches annotation for TCF3: transcription factor 3 (E2A immunoglobulin enhancer binding factors E12/E47)	−1
	8	MORF_TPT1	C4	Neighborhood of TPT1	−1
	9	HSA03010_RIBOSOME	C2	Genes involved in ribosome	−1
	10	AACTTT_UNKNOWN	C3	Genes with promoter regions [-2kb,2kb] around transcription start site containing motif AACTTT. Motif does not match any known transcription factor	−1
HER2+	1	BRCA_ER_POS	C2	Genes whose expression is consistently positively correlated with estrogen receptor status in breast cancer - higher expression is associated with ER-positive tumors	−1
	2	BRCA_ER_NEG	C2	Genes whose expression is consistently negatively correlated with estrogen receptor status in breast cancer - higher expression is associated with ER-negative tumors	−1
	3	module_5	C4	Genes in module_5	1
	4	MORF_TPT1	C4	Neighborhood of TPT1	−1
	5	GNF2_EIF3S6	C4	Neighborhood of EIF3S6	−1
	6	NUCLEUS	C5	Genes annotated by the GO term GO:0005634. A membrane-bounded organelle of eukaryotic cells in which chromosomes are housed and replicated. In most cells, the nucleus contains all of the cell's chromosomes except the organellar chromosomes, and is the site of RNA synthesis and processing. In some species, or in specialized cell types, RNA metabolism or DNA replication may be absent.	−1
	7	MORF_RAD23A	C4	Neighborhood of RAD23A	−1
	8	PENG_GLUTAMINE_DN	C2	Genes downregulated in response to glutamine starvation	−1
	9	GNF2_FBL	C4	Neighborhood of FBL	−1
	10	module_1	C4	Genes in module_1	1

Table 13: set.u.stat.pval.log

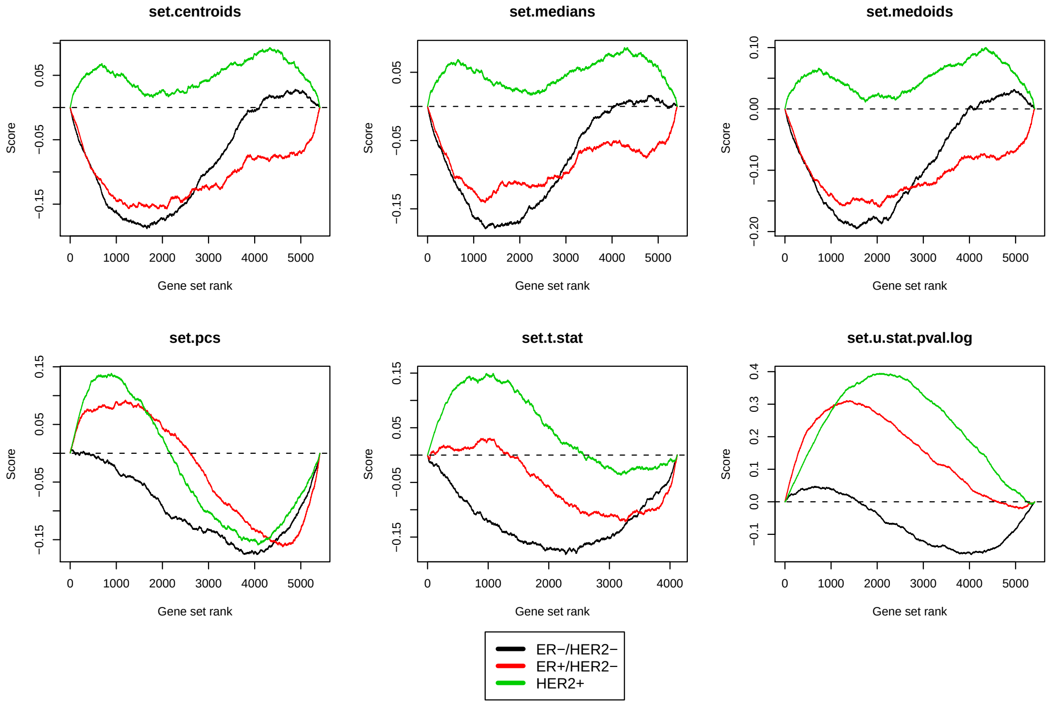

Figure 11: Kolmogorov-Smirnov plots for overlap between gene sets and modules from [24].

Overlap between top genes and gene sets for different classifiers

Tables 14, 15, 16, 17, 18, 19 show the top 10 sets for each classifier, and the p-value for the number of top genes belonging to each of them (Fisher's exact test, one sided). CC is centroid classifier, LR is logistic regression.

Classifier	#	MSigDB set	p-value	matches	set size
CC	1	GNF2_MKI67	$< 1.00\times10^{-40}$	31	47
	2	GNF2_TTK	$< 1.00\times10^{-40}$	29	57
	3	GNF2_CCNA2	$< 1.00\times10^{-40}$	48	99
	4	GNF2_HMMR	$< 1.00\times10^{-40}$	42	78
	5	GNF2_SMC2L1	$< 1.00\times10^{-40}$	26	51
	6	GNF2_CDC20	$< 1.00\times10^{-40}$	46	91
	7	GNF2_ESPL1	$< 1.00\times10^{-40}$	27	58
	8	GNF2_H2AFX	$< 1.00\times10^{-40}$	24	54
	9	GNF2_RRM2	$< 1.00\times10^{-40}$	32	68
	10	chr1q11	2.32×10^{-6}	2	4
SVM	1	chr7q12	6.23×10^{-4}	1	1
	2	chr3q11	1.00	0	8
	3	chrxq	1.00	0	2
	4	BYSTRYKH_RUNX1_TARGETS_GLOCUS	8.06×10^{-3}	1	13
	5	TESTIS_EXPRESSED_GENES	7.28×10^{-7}	4	107
	6	chr22q	1.00	0	6
	7	REGULATION_OF_G_PROTEIN_COUPLED_RECEPTOR_PROTEIN_SIGNALING_PATHWAY	4.28×10^{-4}	2	48
	8	chr11p14	1.00	0	20
	9	TERCPATHWAY	1.00	0	15
	10	chr1q41	2.02×10^{-4}	2	33
LR	1	chr3q11	1.00	0	8
	2	chr22q	1.00	0	6
	3	TERCPATHWAY	1.00	0	15
	4	chrxq	1.00	0	2
	5	BYSTRYKH_RUNX1_TARGETS_GLOCUS	8.06×10^{-3}	1	13
	6	HSA00130_UBIQUINONE_BIOSYNTHESIS	1.00	0	8
	7	chr20p	1.00	0	2
	8	chr1q41	1.29×10^{-6}	3	33
	9	chr3q12	1.00	0	23
	10	BETA_TUBULIN_BINDING	1.00	0	12

Table 14: set.centroids

Classifier	#	MSigDB set	p-value	matches	set size
CC	1	GNF2_MKI67	$< 1.00\times10^{-40}$	31	47
	2	GNF2_TTK	$< 1.00\times10^{-40}$	29	57
	3	chr1q11	2.32×10^{-6}	2	4
	4	GNF2_CCNA2	$< 1.00\times10^{-40}$	48	99
	5	GNF2_HMMR	$< 1.00\times10^{-40}$	42	78
	6	GNF2_SMC2L1	$< 1.00\times10^{-40}$	26	51
	7	GNF2_CDC20	$< 1.00\times10^{-40}$	46	91
	8	GNF2_ESPL1	$< 1.00\times10^{-40}$	27	58
	9	GNF2_H2AFX	$< 1.00\times10^{-40}$	24	54
	10	GNF2_RRM2	$< 1.00\times10^{-40}$	32	68
SVM	1	chr3q11	1.00	0	8
	2	chr4p14	1.00	0	18
	3	TERCPATHWAY	1.00	0	15
	4	chr20p	1.00	0	2
	5	SENSORY_PERCEPTION_OF_ TASTE	1.00	0	5
	6	chr18q22	1.00	0	13
	7	chrxq	1.00	0	2
	8	TESTIS_EXPRESSED_GENES	7.28×10^{-7}	4	107
	9	AS3_FIBRO_C4	2.64×10^{-2}	1	43
	10	chr1q41	2.02×10^{-4}	2	33
LR	1	chr3q11	1.00	0	8
	2	TERCPATHWAY	1.00	0	15
	3	SENSORY_PERCEPTION_OF_ TASTE	1.00	0	5
	4	chr20p	1.00	0	2
	5	CAPROLACTAM_DEGRADATION	4.97×10^{-3}	1	8
	6	chr22q	1.00	0	6
	7	chr4p14	1.00	0	18
	8	CHEOK_LDMTX_MP_DN	1.00	0	4
	9	IRINOTECAN_PATHWAY_PHAR-MGKB	1.00	0	21
	10	chr15q12	3.73×10^{-3}	1	6

Table 15: set.medoids

Classifier	#	MSigDB set	p-value	matches	set size
CC	1	GNF2_MKI67	$< 1.00\times10^{-40}$	31	47
	2	chr1q11	2.32×10^{-6}	2	4
	3	P21_P53_EARLY_DN	1.71×10^{-30}	11	21
	4	GNF2_TTK	$< 1.00\times10^{-40}$	29	57
	5	GNF2_HMMR	$< 1.00\times10^{-40}$	42	78
	6	GNF2_CDC20	$< 1.00\times10^{-40}$	46	91
	7	GNF2_CCNA2	$< 1.00\times10^{-40}$	48	99
	8	GNF2_SMC2L1	$< 1.00\times10^{-40}$	26	51
	9	GNF2_ESPL1	$< 1.00\times10^{-40}$	27	58
	10	GNF2_RRM2	$< 1.00\times10^{-40}$	32	68
SVM	1	BYSTRYKH_RUNX1_TARGETS_ GLOCUS	8.06×10^{-3}	1	13
	2	SENSORY_PERCEPTION_OF_ TASTE	1.00	0	5
	3	chr3q11	1.00	0	8
	4	chr15q25	1.00	0	41
	5	chr3q12	1.42×10^{-2}	1	23
	6	chr18q	6.23×10^{-4}	1	1
	7	chr7q12	6.23×10^{-4}	1	1
	8	HSA00472_D_ARGININE_AND_D_ ORNITHINE_METABOLISM	1.00	0	1
	9	module_496	1.00	0	9
LR	1	SENSORY_PERCEPTION_OF_ TASTE	1.00	0	5
	2	chr3q11	1.00	0	8
	3	chr3q12	1.00	0	23
	4	BYSTRYKH_RUNX1_TARGETS_ GLOCUS	8.06×10^{-3}	1	13
	5	chr15q25	1.00	0	41
	6	HSA00472_D_ARGININE_AND_D_ ORNITHINE_METABOLISM	1.00	0	1
	7	CHEOK_LDMTX_MP_DN	1.00	0	4
	8	CGTCTTA,MIR-208	1.00	0	11
	9	module_571	1.00	0	25
	10	HSA00130_UBIQUINONE_BIOSYN- THESIS	1.00	0	8

Table 16: set.medians

Classifier	#	MSigDB set	p-value	matches	set size
CC	1	STEMCELL_NEURAL_UP	$< 1.00\times10^{-40}$	93	2712
	2	module_54	$< 1.00\times10^{-40}$	76	388
	3	STEMCELL_EMBRYONIC_UP	$< 1.00\times10^{-40}$	63	1964
	4	module_98	$< 1.00\times10^{-40}$	44	729
	5	TARTE_PLASMA_BLASTIC	$< 1.00\times10^{-40}$	61	585
	6	module_198	$< 1.00\times10^{-40}$	42	564
	7	module_52	$< 1.00\times10^{-40}$	65	773
	8	module_3	$< 1.00\times10^{-40}$	42	723
	9	HOFFMANN_BIVSBII_BI_TABLE2	$< 1.00\times10^{-40}$	55	329
	10	module_252	$< 1.00\times10^{-40}$	37	443
SVM	1	chr12q15	2.14×10^{-4}	2	34
	2	HSA00472_D_ARGININE_AND_D_ ORNITHINE_METABOLISM	1.00	0	1
	3	chr2p	1.00	0	13
	4	AS3_HEK293_UP	2.54×10^{-5}	2	12
	5	CENTROSOME_CYCLE	1.00	0	12
	6	EXTRINSICPATHWAY	3.16×10^{-7}	3	21
	7	CIS_RESIST_LUNG_UP	1.00	0	20
	8	module_407	7.77×10^{-7}	3	28
	9	ETCPATHWAY	1.00	0	14
	10	HSA00791_ATRAZINE_DEGRADA- TION	1.00	0	10
LR	1	chr12q15	2.14×10^{-4}	2	34
	2	chr1q41	1.29×10^{-6}	3	33
	3	chr2q31	1.33×10^{-9}	5	73
	4	chr12q14	1.00	0	41
	5	ARGGGTTAA_UNKNOWN	5.36×10^{-3}	2	173
	6	GNF2_TTN	1.66×10^{-4}	2	30
	7	chr14q22	2.88×10^{-2}	1	47
	8	HINATA_NFKB_DN	1.20×10^{-8}	4	39
	9	chr10q23	1.00	0	86
	10	V$PXR_Q2	1.50×10^{-14}	10	314

Table 17: set.pcs

Classifier	#	MSigDB set	p-value	matches	set size
CC	1	GNF2_CCNA2	$< 1.00\times10^{-40}$	48	99
	2	module_54	$< 1.00\times10^{-40}$	76	388
	3	GNF2_PCNA	$< 1.00\times10^{-40}$	45	104
	4	GNF2_CDC20	$< 1.00\times10^{-40}$	46	91
	5	GNF2_HMMR	$< 1.00\times10^{-40}$	42	78
	6	GNF2_RRM1	$< 1.00\times10^{-40}$	44	144
	7	GNF2_MKI67	$< 1.00\times10^{-40}$	31	47
	8	GNF2_CDC2	$< 1.00\times10^{-40}$	49	99
	9	GNF2_TTK	$< 1.00\times10^{-40}$	29	57
	10	GNF2_CENPF	$< 1.00\times10^{-40}$	46	97
SVM	1	HOMOPHILIC_CELL_ADHESION	1.00	0	35
	2	TESTIS_EXPRESSED_GENES	8.17×10^{-7}	4	107
	3	YTCCCRNNAGGY_UNKNOWN	1.16×10^{-3}	2	77
	4	REGULATION_OF_G_PROTEIN_ COUPLED_RECEPTOR_PROTEIN_ SIGNALING_PATHWAY	4.54×10^{-4}	2	48
	5	AD12_ANY_DN	1.00	0	35
	6	YGACNNYACAR_UNKNOWN	4.04×10^{-5}	3	100
	7	chr2q35	3.71×10^{-2}	1	59
	8	REGULATION_OF_ANATOMICAL_ STRUCTURE_MORPHOGENESIS	1.00	0	33
	9	chr5q34	1.97×10^{-2}	1	31
LR	1	TESTIS_EXPRESSED_GENES	4.95×10^{-5}	3	107
	2	CACGTTT,MIR-302A	2.55×10^{-4}	2	36
	3	chrxq28	6.93×10^{-2}	1	112
	4	LEE_TCELLS3_UP	8.23×10^{-2}	1	134
	5	HOMOPHILIC_CELL_ADHESION	1.00	0	35
	6	ADIPOGENESIS_HMSC_CLASS8_DN	3.71×10^{-2}	1	59
	7	AD12_ANY_DN	1.00	0	35
	8	YTCCCRNNAGGY_UNKNOWN	1.16×10^{-3}	2	77
	9	module_274	4.51×10^{-10}	6	134
	10	ADIP_VS_PREADIP_UP	5.74×10^{-4}	2	54

Table 18: set.t.stat

Classifier	#	MSigDB set	p-value	matches	set size
CC	1	MORF_TPT1	1.40×10^{-1}	1	243
	2	STRUCTURAL_CONSTITUENT_ OF_RIBOSOME	8.92×10^{-2}	1	150
	3	HSA03010_RIBOSOME	7.26×10^{-2}	1	121
	4	module_117	$< 1.00\times10^{-40}$	36	1059
	5	GCM_TPT1	4.17×10^{-3}	2	152
	6	RIBOSOMAL_PROTEINS	2.18×10^{-4}	3	182
	7	MORF_JUND	8.81×10^{-2}	1	148
	8	MORF_ACTG1	1.17×10^{-3}	3	325
	9	GGGAGGRR_V$MAZ_Q6	$< 1.00\times10^{-40}$	55	2670
	10	module_83	4.47×10^{-12}	10	559
SVM	1	chr20q13	5.38×10^{-11}	7	192
	2	module_27	1.75×10^{-19}	15	608
	3	ALCALAY_AML_NPMC_DN	3.24×10^{-11}	8	307
	4	LI_FETAL_VS_WT_KIDNEY_DN	7.64×10^{-4}	3	280
	5	GNF2_DAP3	1.31×10^{-1}	1	225
	6	IGLESIAS_E2FMINUS_UP	2.46×10^{-13}	9	275
	7	chr1q41	2.02×10^{-4}	2	33
	8	module_306	1.00	0	45
LR	1	chr20q13	5.38×10^{-11}	7	192
	2	GGGAGGRR_V$MAZ_Q6	$< 1.00\times10^{-40}$	62	2670
	3	module_27	8.24×10^{-23}	17	608
	4	ALCALAY_AML_NPMC_DN	9.95×10^{-4}	3	307
	5	LI_FETAL_VS_WT_KIDNEY_DN	3.10×10^{-8}	6	280
	6	GNF2_DAP3	1.00	0	225
	7	module_306	1.00	0	45
	8	TGGAAA_V$NFAT_Q4_01	$< 1.00\times10^{-40}$	62	2193
	9	chr2p11	3.97×10^{-2}	1	65
	10	chr1q41	1.29×10^{-6}	3	33

Table 19: set.u.stat.pval.log

References

[1] A. J. Matlin, F. Clark, and C. W. J. Smith. Understanding alternative splicing: towards a cellular code. *Nat. Rev. Mol. Cell. Biol.*, 6:386–398, 2005.

[2] R Development Core Team. *R: A Language and Environment for Statistical Computing.* R Foundation for Statistical Computing, Vienna, Austria, 2008. URL http://www.R-project.org. ISBN 3-900051-07-0.

[3] R. C. Gentleman, V.J. Carey, D. M. Bates, et al. Bioconductor: Open software development for computational biology and bioinformatics. *Genome Biology*, 5:R80, 2004.

[4] L. Gautier, L. Cope, B. M. Bolstad, and R. A. Irizarry. affy—analysis of affymetrix genechip data at the probe level. *Bioinformatics*, 20(3):307–315, 2004.

[5] B. M. Bolstad. *preprocessCore: A collection of pre-processing functions*, 2009. R package version 1.6.0.

[6] M. Morgan, S. Falcon, and R. Gentleman. *GSEABase: Gene set enrichment data structures and methods*, 2009. R package version 1.6.0.

[7] M. Carlson, S. Falcon, H. Pages, and N. Li. *hgu133a.db: Affymetrix Human Genome U133 Set annotation data (chip hgu133a)*, 2009. R package version 2.2.5.

[8] L. J. van 't Veer, H. Dai, M. J. van de Vijver, Y. D. He, A. A. M. Hart, M. Mao, H. L. Peterse, K. van der Kooy, M. J. Marton, A. T. Witteveen, G. J. Schreiber, R. M. Kerkhoven, C. Roberts, P. S. Linsley, R. Bernards, and S. H. Friend. Gene expression profiling predicted clinical outcome of breast cancer. *Nature*, 415:530–536, 2002.

[9] M. Carlson, S. Falcon, H. Pages, and N. Li. *GO.db: A set of annotation maps describing the entire Gene Ontology*, 2009. R package version 2.2.11.

[10] R. Tibshirani, T. Hastie, B. Narasimhan, and G. Chu. Class Prediction by Nearest Shrunken Centroids, with Applications to DNA Microarrays. *Stat. Sci.*, 18:104–117, 2003.

[11] T. Hastie, R. Tibshirani, B. Narasimhan, and G. Chu. *pamr: Pam: prediction analysis for microarrays*, 2009. R package version 1.42.0.

[12] A. Karatzoglou, A. Smola, K. Hornik, and A. Zeileis. kernlab – an S4 package for kernel methods in R. *Journal of Statistical Software*, 11(9):1–20, 2004. URL http://www.jstatsoft.org/v11/i09/.

[13] M. J. van de Vijver, Y. D. He, L. J. van 't Veer, H. Dai, A. A. M. Hart, D. W. Voskuil, G. J. Schreiber, J. L. Peterse, C. Roberts, M. J. Marton, M. Parrish, D. Atsma, A. Witteveen, A. Glas, L. Delahaye, T. van der Velde, H. Bartelink, S. Rodenhuis, E. T. Rutgers, S. H. Friend, and R. Bernards. A gene-expression signature as a predictor of survival in breast cancer. *New Engl. J. Med.*, 347:1999–2009, 2002.

[14] R. J. Tibshirani and B. Efron. Pre-validation and inference in microarrays. *Statist. Appl. Genet. Mol. Biol.*, 1:1, 2002.

[15] G. J. McLachlan, K.-A. Do, and C. Ambroise. *Analyzing Microarray Gene Expression Data.* Wiley Interscience, 2004.

[16] J. A. Hanley and B. J. McNeil. The Meaning and Use of the Area under a Receiver Operating Characteristic (ROC) Curve. *Radiology*, 143:29–36, 1982.

[17] D. W. Mehlman, U. L. Sheperd, and D. A. Kelt. Bootstrapping Principal Components Analysis: A Comment. *Ecology*, 76:640–643, 1995.

[18] J. O. Ramsay and B. W. Silverman. *Functional Data Analysis.* Springer, 2nd edition, 2006.

[19] E. Bair and R. Tibshirani. Semi-Supervised Methods to Predict Patient Survival from Gene Expression Data. *PLoS Biology*, 2:0511–0522, 2004.

[20] V. K. Mootha, C. M. Lindgren, K.-F. Eriksson, A. Subramanian, S. Sihag, J. Lehar, P. Puigserver, E. Carlsson, M. Ridderstråle, E. Laurila, N. Houstis, M. J. Daly, N. Patterson, J. P. Mesirov, T. R. Golub, P. Tamayo, B. Spiegelman, E. S. Lander, J. N. Hirschhorn, D. Altshuler, and L. C. Groop. PGC-1α-responsive genes involved in oxidative phosphorylation are coordinately downregulated in human diabetes. *Nat. Genet.*, 34:267–273, 2003.

[21] A. Subramanian, P. Tamayo, V. K. Mootha, S. Mukherjee, B. L. Ebert, M. A. Gillette, A. Paulovich, S. L. Pomeroy, T. R. Golub, E. S. Lander, and J. P. Mesirov. Gene set enrichment analysis: A knowledge-based approach for interpreting genome-wide expression profiles. *Proc. Natl. Acad. Sci.*, 102:15545–15550, 2005.

[22] M. Hollander and D. A. Wolfe. *Nonparametric Statistical Methods.* Wiley-Interscience, 2nd edition, 1999.

[23] J. W. Pratt and J. D. Gibbons. *Concepts of Nonparametric Theory.* Springer-Verlag, 1981.

[24] C. Desmedt, B. Haibe-Kains, P. Wirapati, M. Buyse, D. Larsimont, G. Bontempi, M. Delorenzi, M. Piccart, and C. Sotiriou. Biological processes associated with breast cancer clinical outcome depend on the molecular subtypes. *Clin. Cancer Res.*, 14:5158–5165, 2008.

[25] F. Leisch. FlexMix: A general framework for finite mixture models and latent class regression in R. *J. Stat. Soft.*, 11:1–18, 2004. URL `http://www.jstatsoft.org/v11/i08/`.
